# A memristor-based energy-efficient compressed sensing accelerator with hardware–software co-optimization for edge computing

**DOI:** 10.1093/nsr/nwaf499

**Published:** 2025-11-13

**Authors:** Yunrui Jiao, Han Zhao, Jianshi Tang, Yanze Zhou, Ruofei Hu, Haochen Jiang, Xingchu Li, Jingyuan Huang, Biao Sun, Wen Sun, Bin Gao, He Qian, Huaqiang Wu

**Affiliations:** School of Integrated Circuits, Beijing Advanced Innovation Center for Integrated Circuits, BNRist, Tsinghua University, Beijing 100084, China; School of Integrated Circuits, Beijing Advanced Innovation Center for Integrated Circuits, BNRist, Tsinghua University, Beijing 100084, China; School of Integrated Circuits, Beijing Advanced Innovation Center for Integrated Circuits, BNRist, Tsinghua University, Beijing 100084, China; School of Integrated Circuits, Beijing Advanced Innovation Center for Integrated Circuits, BNRist, Tsinghua University, Beijing 100084, China; School of Integrated Circuits, Beijing Advanced Innovation Center for Integrated Circuits, BNRist, Tsinghua University, Beijing 100084, China; School of Integrated Circuits, Beijing Advanced Innovation Center for Integrated Circuits, BNRist, Tsinghua University, Beijing 100084, China; School of Integrated Circuits, Beijing Advanced Innovation Center for Integrated Circuits, BNRist, Tsinghua University, Beijing 100084, China; School of Integrated Circuits, Beijing Advanced Innovation Center for Integrated Circuits, BNRist, Tsinghua University, Beijing 100084, China; School of Electrical and Information Engineering, Tianjin University, Tianjin 300072, China; School of Integrated Circuits, Beijing Advanced Innovation Center for Integrated Circuits, BNRist, Tsinghua University, Beijing 100084, China; School of Integrated Circuits, Beijing Advanced Innovation Center for Integrated Circuits, BNRist, Tsinghua University, Beijing 100084, China; School of Integrated Circuits, Beijing Advanced Innovation Center for Integrated Circuits, BNRist, Tsinghua University, Beijing 100084, China; School of Integrated Circuits, Beijing Advanced Innovation Center for Integrated Circuits, BNRist, Tsinghua University, Beijing 100084, China

**Keywords:** compressed sensing, memristor chip, hardware–software co-optimization, computing-in-memory, edge computing

## Abstract

Compressed sensing (CS), a revolutionary signal processing technique enabling sub-Nyquist sampling, has become integral to reduce hardware cost and energy consumption in diverse applications. However, with the exponential growth of data, traditional Si complementary metal-oxide semiconductor (CMOS)-based hardware implementations face significant challenges, including the von Neumann bottleneck in energy efficiency and computing latency. In this work, we propose a memristor-based CS accelerator (memCS) that leverages computing-in-memory (CIM) to eliminate the data movement overhead. Using a fully integrated 128 Kb memristor chip, we systematically analyze the impact of non-ideal device characteristics, and further propose a hardware–software co-optimization framework that integrates the measurement matrix modification (MMM) and sparsity enhancement (SE) strategies, leading to significantly enhanced noise robustness and reconstruction accuracy. Our memCS eventually achieves a near-software peak signal-to-noise ratio (PSNR) of 31.11 dB and a high accuracy of 94.2% in the image classification task on the ImageNet dataset. Benchmarking results further demonstrate that the memCS greatly outperforms state-of-the-art CMOS hardware by achieving 11.22 times speedup and 30.46 times energy savings, thereby providing a scalable solution for energy-efficient edge computing applications.

## Teaser

This work presents a hardware–software co-optimization framework for a memristor-based energy-efficient compressed sensing accelerator, enabling 11.22× speedup and 30.46× energy savings.

## INTRODUCTION

**Figure 1.**
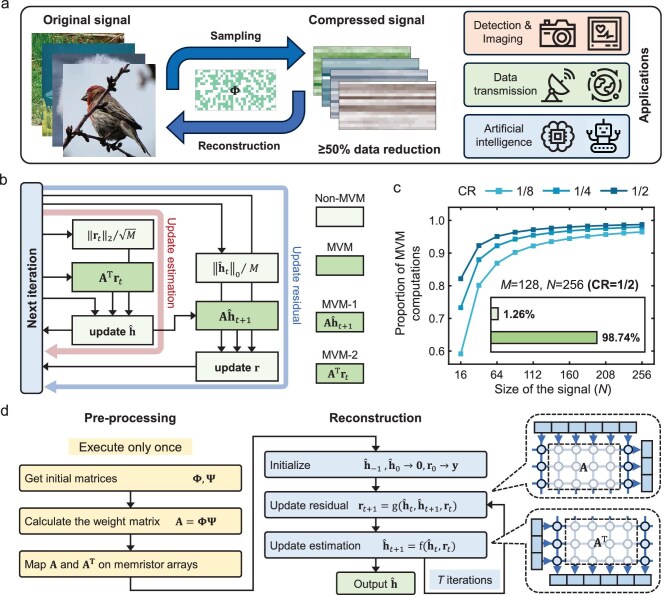
Overview of compressed sensing and memristor-based CS accelerator (memCS). (a) Illustration of the fundamental steps in CS and its potential applications. ${\bf{\Phi}}$, the measurement matrix. (b) Stepwise computing in a round of iterative update in the AMP algorithm. The MVMs in the update of residual and estimation are denoted as MVM-1 (**Aĥ***_t_*_+1_) and MVM-2 (**A**^T^**r***_t_*), respectively. **A**, the weight matrix, i.e. the multiplication of the measurement matrix ${\bf{\Phi}}$ and the sparse transform matrix ${\bf{\Psi}}$. (c) Proportion of MVM computations in the AMP algorithm with different signal sizes. CR, compression ratio (M/N); M and N, sizes of the original and compressed signals. (d) Reconstruction dataflow of memCS using the AMP algorithm, with pre-processing steps and iterative updates of the estimation and residual.

With the fast development of artificial intelligence (AI) and the Internet of Things (IoT), a large number of sensor nodes have been constructed to sample signals and store them at the edge devices [1,2]. Compressed sensing (CS) is a revolutionary signal processing technique that allows accurate signal reconstruction using significantly fewer samples [3]. The CS framework, as illustrated in Fig. 1a, encompasses both the fundamental process of signal sampling and reconstruction, as well as its diverse applications. This paradigm has enabled a broad range of implementations, including image detection and reconstruction [4–6], energy-efficient data transmission [7,8] and AI [9,10]. CS conventionally employs random sub-sampling procedures at the edge, whereupon the resultant compressed signals are transmitted to remote computing platforms for subsequent reconstruction [11,12]. Nevertheless, recent explosive growth of data imposes significant challenges to energy-efficient implementation of CS, since digital computers based on conventional von Neumann architecture with physically separated memory and processing units face severe bottlenecks on transfer latency and energy consumption [13,14].

To address the above challenges, the memristor array, with the advantages of analog computing-in-memory (CIM), can efficiently perform matrix–vector multiplication (MVM) in parallel [15–25], which serves as the core computation in the reconstruction stages of CS. In this work, we present a memristor-based CS accelerator (memCS), which consists of two fully integrated 128 Kb HfO_2_-based memristor chips. The approximate message passing (AMP) algorithm is implemented due to its good compatibility with memristor chips [26,27]. As shown in Fig. 1b, the AMP algorithm can be decomposed into MVM operations with O(*N × M*) complexity and non-MVM operations with O(*N* + *M*) complexity. Figure 1c further demonstrates that MVM operations constitute up to 98.74% of total computing operations for typical parameters (*M* = 128, *N* = 256), confirming the significant acceleration potential of memCS through a CIM paradigm. The procedural framework for memCS-based signal reconstruction using the AMP algorithm is presented in Fig. 1d. During the preprocessing phase, the product of the measurement matrix and the sparse transform matrix, **A** = ${\bf{\Phi \Psi}}$, is computed. Subsequently, both matrices **A** and **A**^T^ are mapped onto the memristor chip, which facilitates *in situ* acceleration of MVMs required for signal updating during the reconstruction phase. For a specific application scenario, ${\bf{\Phi}}$ and ${\bf{\Psi}}$ are typically fixed, necessitating the once-only execution of the aforementioned steps. In the reconstruction phase, the estimation in the sparse domain ${\mathrm{\bf \hat{h}}}$ is initialized to ${\bf 0} $, while the residual ${\mathrm{\bf r}}$ is initialized to the compressed signal ${\mathrm{\bf y}}$. Through iterative updates of ${\mathrm{\bf \hat{h}}}$ and ${\mathrm{\bf r}}$, the reconstructed signal is obtained. However, during this process, the inherent non-ideal characteristics of the memristor array would inevitably induce computing errors in MVM operations compared to software-based CS implementations [28–30]. These errors may propagate and accumulate progressively throughout the iterative process, potentially degrading the reconstruction accuracy.

Therefore, to mitigate the accuracy loss, in this work, we first conduct a systematic analysis on the impact of the memristor’s non-ideal characteristics, based on which we propose two synergistic hardware–software co-optimization strategies. First, following the mathematical theory on the CS measurement matrix, as well as the hardware characteristics of memristor chip, we develop the measurement matrix modification (MMM) approach to effectively improve the memCS’s robustness. Second, considering the inherent sparsity requirements of CS, we propose the sparsity enhancement (SE) strategy, which introduces a multi-stage discrete wavelet transform tailored to the memristor chip to significantly improve the signal sparsity. Experimental results show that they can reduce MVM computing errors by over 8-fold after 20 iterations (i.e. 40 MVMs), and achieve a remarkable 5.53 dB peak signal-to-noise ratio (PSNR) enhancement in the image reconstruction. Further, we implement a practical CS task on the ImageNet dataset [31], and the memCS achieves near-software PSNR (31.11 dB) and classification accuracy (94.2%). Notably, the system-level performance benchmark reveals that memCS is 11.22 times faster and consumes 30.46 times less energy compared to the state-of-the-art complementary metal-oxide semiconductor (CMOS) implementation. This work has identified an optimized CS solution that is well suited for memristors, effectively enhancing the reconstruction accuracy while achieving significant advantages in energy efficiency and computing speed, thereby providing a scalable solution for high-performance edge computing applications.

## Mathematical analysis of the matrix and algorithm in CS

In the sampling stage of CS, the original signal **x** ∈ ${\mathbb{R}}$*^N^* is multiplied by a matrix ${\bf{\Phi}}$ ∈ ${\mathbb{R}}$*^M×^^N^* to obtain the compressed signal **y** ∈ ${\mathbb{R}}$*^M^* (*M* < *N*). Usually, the original signal ${\mathrm{\bf x}}$ can be represented sparsely as ${\mathrm{\bf h}}$ in a certain transform domain (i.e. **x** = ${\bf{\Psi}}$**h**, ${\bf{\Psi}}$ ∈ ${\mathbb{R}}$*^N×N^*). Thus, the sampling stage can be expressed as Equation (1):


(1) \begin{eqnarray*}
{\mathrm{\bf y}} = {\mathrm{\bf \Phi x}} = {\mathrm{\bf \Phi \Psi h}} = {\mathrm{\bf Ah}}.
\end{eqnarray*}

The reconstruction process is exactly the inverse of the sampling process. CS exploits the sparsity constraints of the signal in the ${\bf{\Psi}}$ domain and obtains ${\mathrm{\bf \hat{h}}}$ (the approximation of ${\mathrm{\bf h}}$) by solving the *l*_0_ norm minimization problem as shown in Equation (2):


(2) \begin{eqnarray*}
{\mathrm{min}}\|{\mathrm{\bf \hat{h}}}{\|}_0\quad {\mathrm{s}}.{\mathrm{t}}.\quad \|{\mathrm{\bf y}} - {\mathrm{\bf A\hat{h}}}{\|}_2 < \varepsilon .
\end{eqnarray*}

For successful signal reconstruction, the matrix **A** must satisfy the restricted isometry property, which specifically requires that the columns of **A** maintain sufficient linear independence to guarantee a unique sparse recovery [32]. Since the sparse transform matrix ${\bf{\Psi}}$ is typically full-rank, the property can be satisfied through careful design of the measurement matrix ${\bf{\Phi}}$.

In the literature, commonly employed algorithms to address the problem in Equation (2) include greedy algorithms [33,34], convex optimization [35–37] and non-convex optimization approaches [38,39]. However, both greedy algorithms and non-convex optimization techniques typically rely on matrix inversion or pseudoinverse computations, which are difficult to be represented as MVM operations, making them unsuitable for implementation on memristor arrays. Fortunately, the AMP algorithm is more favorable for memCS, since its core operations in the iterative update of residual and estimation are both MVM with a fixed matrix. As illustrated in Equations (3) and (4):


(3) \begin{eqnarray*}
{{\mathrm{\bf \hat{h}}}}_{t + 1} = {{\mathrm{\eta }}}_t\left( {{{\mathrm{\bf A}}}^{\mathrm{T}}{{\mathrm{\bf r}}}_t + {{{\mathrm{\bf \hat{h}}}}}_t} \right)
\end{eqnarray*}

(4) \begin{eqnarray*}
\ {{\mathrm{\bf r}}}_{t + 1} = {\mathrm{\bf y}} - {\mathrm{\bf A}}{{\mathrm{\bf \hat{h}}}}_{t + 1} + \frac{N}{M}\ {{\mathrm{\bf r}}}_t\ \left\langle {{\mathrm{\eta }}_t^{\prime}\left( {{{\mathrm{\bf A}}}^{\mathrm{T}}{{\mathrm{\bf r}}}_t + {{{\mathrm{\bf \hat{h}}}}}_t} \right)} \right\rangle .
\end{eqnarray*}

In these formulations, ⟨⋅⟩ denotes the averaging operation across all elements of the input vector, while ${{\mathrm{\eta }}}_t$(⋅) denotes a thresholding function with threshold value *t* that operates element-wise on vectors to maintain signal sparsity. In this work, the soft thresholding function is adopted as the thresholding mechanism. The expressions of ${{\mathrm{\eta }}}_t$(⋅) and its derivative ${\mathrm{\eta }}_t^{\prime}$(⋅) are given in Equations (5) and (6):


(5) \begin{eqnarray*}
{{\mathrm{\eta }}}_t\!\left( x \right) &=& {\mathrm{sgn}}\!\left( x \right) \cdot \max\! \left( {\left| x \right| - t,\ 0} \right)\\
&=& \left\{ {\begin{array}{c@{\quad}c} 0, & \left| x \right| < t\\
x - t, & x \ge t\\ x + t, & x \le - t \end{array}} \right.
\end{eqnarray*}

(6) \begin{eqnarray*}
{\mathrm{\eta }}_t^{\prime}\!\left( x \right) = \frac{{1 + {\mathrm{sgn}}\!\left( {\left| x \right| - t} \right)}}{2} = \left\{ {\begin{array}{c@{\quad}c} 0, & \left| x \right| < t\\ 1, & \left| x \right| \ge t \end{array}} \right..
\end{eqnarray*}

Under this specification, Equations (3) and (4) can be decomposed into the procedural flowchart depicted in Fig. 1b. As demonstrated in Fig. 1c, MVM operations constitute the dominant computational burden, confirming that the AMP algorithm implemented on memCS can yield significant acceleration by taking advantage of the CIM of memristors.

## Hardware implementation and characterizations of memCS

**Figure 2.**
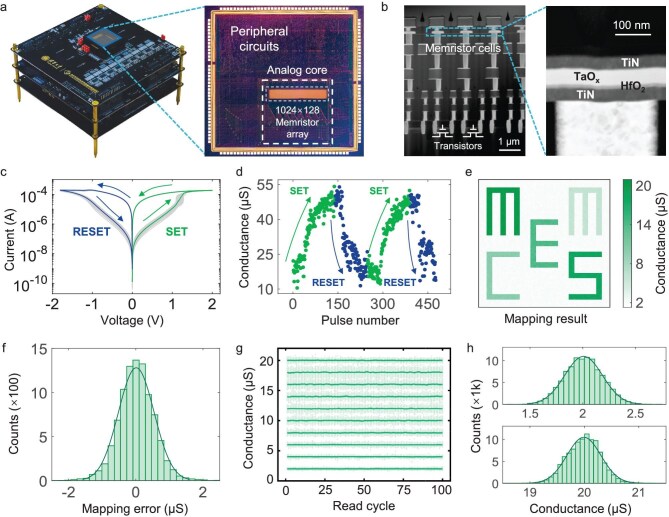
Characteristics of the memristor chip. (a) Photographs of the test system and the 128 Kb memristor chip. (b) Transmission electron microscopy (TEM) images of the memristor chip, showing the device structure of TiN/TaO_x_/HfO_2_/TiN. (c) Typical DC I–V characteristics, illustrating the SET and RESET processes. The gray lines represent 50 switching cycles and the colored lines represent the average value. (d) Analog switching characteristics under SET and RESET voltage pulse sequences. (e) Mapping results of the ‘MEMCS’ pattern on the memristor array with different conductance levels. (f) Statistical analysis of the mapping error between the mapping results and target values. (g) Fluctuations of read conductance values from 100 memristors at each conductance level over 100 read cycles. The light and dark green lines represent the read conductance of individual device and the average values, respectively. (h) Distribution of the read conductance in the lowest and highest conductance levels.

In the implementation of memCS, two memristor chips are used as the hardware accelerator. As shown in Fig. 2a, each chip consists of an analog core and peripheral controlling circuits. The analog core comprises a 128 Kb one-transistor-one-resistor (1T1R) memristor array with 1024 columns and 128 rows. The memristor device has a typical material stack of TiN/HfO_2_/TaO_x_/TiN (Fig. 2b) [40,41], with its direct current–voltage (DC I–V) characteristics in Fig. 2c indicating reliable resistive switching. By applying a series of SET and RESET voltage pulses, the devices can be programmed to different conductance levels, as shown in Fig. 2d, which shows good analog resistive switching characteristics. To demonstrate the mapping precision, as depicted in Fig. 2e and f, we have mapped a letter pattern ‘MEMCS’ onto the 64 × 64 memristor array using 10 different conductance states ranging from 2 to 20 μS, and subsequently analyzed the deviation between the target and mapped conductance values. Statistical results indicate that the programming error approximately conforms to a Gaussian distribution with a standard deviation (SD) level of 0.5 μS. In addition, the read noise, indicating the conductance fluctuation over time, is illustrated in Fig. 2g and h. The noise distribution in each conductance level also follows a Gaussian distribution. The empirical characterization of these device-level non-idealities lays the foundation for our subsequent analysis of error propagation and accumulation throughout the iterative reconstruction process. More details about the device characterization are provided in Note S1.

**Figure 3.**
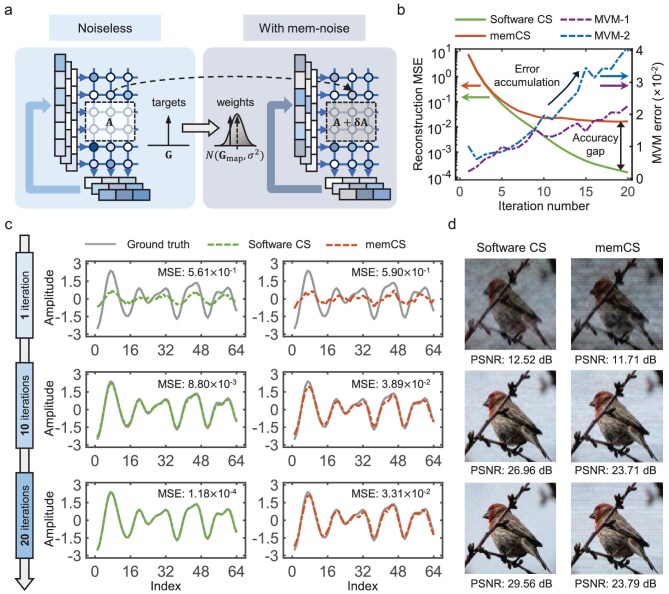
Analysis of error propagation and accumulation in memCS. (a) Schematic illustration of error propagation and accumulation during the memCS reconstruction process due to memristor non-idealities. **G**_map_, post-mapping conductance value; σ, SD level of read noise. (b) Illustration of error accumulation in memCS. In this experiment, the MVM error is quantified utilizing mean squared error (MSE) as the metric. (c and d) Comparison of reconstructed signals between software CS and memCS after 1, 10 and 20 iterations in both 1D (c) and 2D (d) scenarios.

Taking the MVM-1 denoted in Fig. 1b as an example, Fig. 3a illustrates the propagation and accumulation of errors arising from memristor noise (including the mapping error and read noise) throughout the memCS reconstruction. Experimentally, after mapping the target value **G** on the memristor device, the actual conductance follows a Gaussian distribution with a deviated mean of **G**_map_ and an SD of *σ*. These non-idealities could severely degrade the computing accuracy of the MVM operations. More seriously, the computing errors would propagate through subsequent processing units, thereby affecting the inputs for the next iterations. Consequently, the MVM computing error between memCS and software CS may be progressively amplified, enlarging the reconstruction accuracy gap after every iteration, as illustrated in Fig. 3b. Experimental results clearly demonstrate the error accumulation effect in both MVMs, with an increasingly significant accuracy discrepancy between the software CS and memCS, ultimately reaching two orders of magnitude difference after 20 iterations.

Experimental reconstruction results of 1D vectors and 2D images with the baseline configured memCS are illustrated in Fig. 3c and d, further demonstrating its obvious accuracy loss. In the 1D scenario (Fig. 3c), following 20 iterations, the software CS reconstruction results almost perfectly replicate the ground truth, achieving a low MSE of 1.18 × 10^−4^, whereas memCS continues to exhibit significant deviations, with a much higher MSE of 4.38 × 10^−2^. In the 2D scenario (Fig. 3d), the original image pattern gradually becomes more distinct as the iteration count increases. Relative to the software CS, the memCS-reconstructed image exhibits conspicuous artifacts (traverse striping resulted from column-wise processing of the image), which substantially degrade the visual quality. This results in a progressively increasing accuracy loss, reaching 5.77 dB after 20 iterations. In conclusion, the mitigation of error accumulation effect arising from memristor noise is crucial for enabling memCS implementations to achieve high reconstruction fidelity comparable to software-based counterparts. It is critical to develop feasible optimization strategies for the accuracy improvement of memCS.

## Hardware–software co-optimization for memCS: MMM and SE

**Figure 4.**
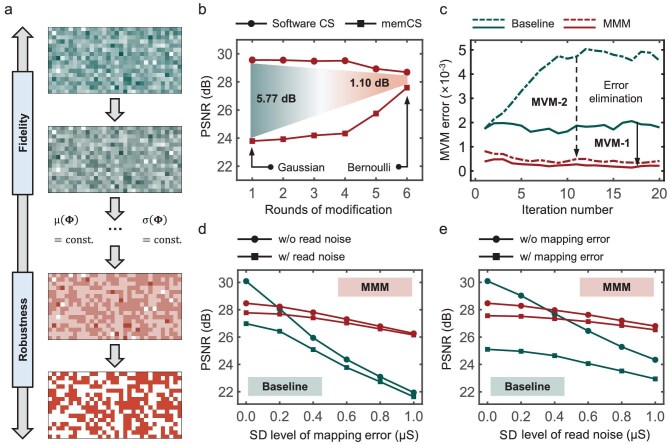
Implementation and validation of the MMM strategy. (a) Illustration of MMM: reducing quantization bits while maintaining the CS mathematical constrains that the mean and SD value of the measurement matrix should remain constant. const, constant. (b) Comparison of the image reconstruction accuracy between software CS and memCS under different rounds of modification. (c) Comparison of the MVM computing errors across iterations with and without MMM. The solid line and the dotted line denote MVM-1 and MVM-2, respectively. (d and e) Comparison of the image reconstruction accuracy of memCS under varying levels of memristor noise [mapping error (d) and read noise (e)] with and without MMM. It is noted that in Fig. 4d and e, and Fig. 5e and f, we set the SD value to 0.5 μS, based on our experimentally measured device statistics (Fig. 2f for mapping error and Fig. S2 for read noise). w/o, without; w/, with.

Following the above error propagation analysis, we first propose the MMM strategy to enhance the robustness of memCS against non-ideal characteristics of memristors. For the Gaussian measurement matrix commonly used in CS, we found that while it exhibits good software reconstruction accuracy, its continuous element distribution is not well suited to memristor characteristics. Specifically, when programming Gaussian matrices on memristor chips, intermediate memristor conductance states are extensively utilized to map the elements. However, these intermediate conductance states typically exhibit larger read noise [42], leading to more severe MVM computing errors. Therefore, we propose the MMM strategy, which optimizes the element distribution of measurement matrices to reduce dependence on unstable intermediate conductance states, thereby improving the overall robustness of memCS. As shown in Fig. 4a, the core step of the MMM strategy is based on a constrained quantization process that reduces bit precision to enable programming the elements to more stable conductance states while strictly following the statistical constraints of CS, which preserves the mean and SD of the matrix through translation and scaling operations. Specifically, the constrained quantization process can be formulated as Equation (7), where the ${\bf{\Phi}}$ and ${\bf{\Phi}}$_MMM_ denote the measurement matrix before and after a round of MMM, Q_MMM_(⋅) represents the quantization operator, *α* and *β* are translation and scaling parameters, and μ(⋅) and σ(⋅) stand for the mean and SD of all elements in the matrix. After obtaining the quantization result Q_MMM_(${\bf{\Phi}}$), we set *β* = σ(${\bf{\Phi}}$)/σ(Q_MMM_(${\bf{\Phi}}$)) and *α* = μ(${\bf{\Phi}}$) − *β*$\cdot $μ(Q_MMM_(${\bf{\Phi}}$)) so that ${\bf{\Phi}}$_MMM_ preserves the same mean and SD as ${\bf{\Phi}}$. It can be further derived that with these adjustments, ${\bf{\Phi}}$_MMM_ maintains the restricted isometry property of CS, ensuring successful reconstruction and improved reconstruction robustness.


(7) \begin{eqnarray*}
{{\mathrm{\bf \Phi }}}_{{\mathrm{MMM}}} &=& \beta \cdot {{\mathrm{Q}}}_{{\mathrm{MMM}}}\!\left( {\mathrm{\bf \Phi }} \right) + \alpha ,\ {\mathrm{s}}.{\mathrm{t}}.\ {\mathrm{\mu }}\!\left( {{{\mathrm{\bf \Phi }}}_{{\mathrm{MMM}}}} \right)\\
&=& {\mathrm{\mu }}\!\left( {\mathrm{\bf \Phi }} \right),\ {\mathrm{\sigma }}\!\left( {{{\mathrm{\bf \Phi }}}_{{\mathrm{MMM}}}} \right) = {\mathrm{\sigma }}\!\left( {\mathrm{\bf \Phi }} \right)\!.
\end{eqnarray*}

Image reconstruction results (Fig. 4b) demonstrate that as the number of modification rounds increases, the reconstruction accuracy of software CS decreases while that of memCS increases, indicating that the MMM strategy could lead to a little bit lower fidelity but much higher robustness of the reconstruction matrix. After six rounds of modification, the accuracy gap between memCS and software CS is reduced by 4.67 dB, at which point the Gaussian matrix is modified into a binary Bernoulli matrix. Building upon this, we further examine the evolution of MVM computing errors during the iterative process, with results shown in Fig. 4c. When using the MMM-optimized measurement matrix, computing errors in both MVM-1 and MVM-2 are reduced to below 10^−3^. Compared to the baseline case (without MMM), the computing errors of MVM-1 and MVM-2 with MMM is decreased by 8.44 times and 11.03 times after 20 iterations, respectively, demonstrating an effective mitigation of the error accumulation. To further investigate the memristor noise robustness enhancement by MMM under different noise levels, we simulate the memCS reconstruction under mapping error and read noise with varying SD levels based on our large amount of device-level experimental results, as presented in Fig. 4d and e. When MMM is not employed (green curves), reconstruction accuracy deteriorates significantly as the noise level increases. In contrast, when using the MMM-optimized measurement matrix (red curves), the PSNR remains stable between ∼26 and 28 dB throughout the entire simulated noise range. This demonstrates that MMM significantly enhances memCS’s tolerance to memristor noise by suppressing MVM computing errors, maintaining stable accuracy under a broader range of memristor conductance fluctuations. In addition, the sensitivity analysis in Note S2 shows that *α* and *β* have a good tolerance to over 10% parameter variations, indicating robust performance against perturbations.

**Figure 5.**
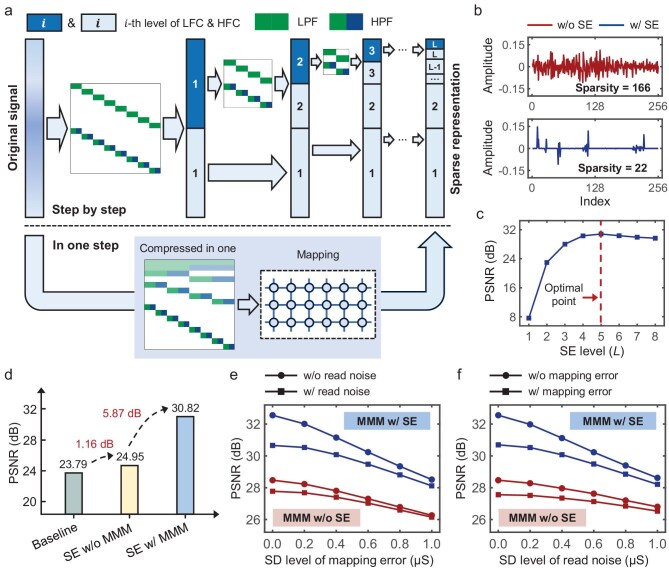
Implementation and validation of sparsity enhancement (SE). (a) Illustration of SE: achieving memristor chip-compatible one-step multi-level DWT through matrix compression. LFC/HFC, low/high frequency components; LPF/HPF, low/high-passed filters. (b) Comparison of signal sparsity with and without SE. w/o, without; w/, with. (c) Image reconstruction accuracy under different SE levels. (d) Synergistic optimization effect of the SE and MMM. (e and f) Comparison of image reconstruction accuracy under varying levels of memristor noise [mapping error (e) and read noise (f)] with and without SE.

Notably, under the optimization of MMM, although the robustness of memCS is enhanced, the reconstruction accuracy remains at a low level (e.g. in Fig. 4d and e, the PSNR is still lower than 30 dB even at low noise levels). Given the mathematical conclusion that the accuracy of CS reconstruction strongly depends on signal sparsity in the transform domain, we leverage the one-step MVM formulation of the multi-level Haar discrete wavelet transform (DWT) [43] to develop a memristor-compatible SE strategy for memCS, and provide a systematic analysis of level selection. As illustrated in Fig. 5a, *L*-level Haar DWT separates information into low-frequency and high-frequency components at different resolutions through *L* steps of recursive decomposition, ultimately preserving signal characteristics with fewer coefficients. However, such multi-level DWT is not suitable for memristor chips, since it requires multiple MVM computations of varying scales, leading to additional energy consumption and latency. According to Stéphane [43], the *L*-level DWT computation can be compressed into a one-step MVM by incorporating the high-pass filters of the first, second, …, *L*th level and the low-pass filter of the *L*th level into a single matrix **W***_L_*, as shown in Equation (8). Furthermore, it can be derived that (**W***_L_*)^−1^ = (**W***_L_*)^T^, which implies that by setting ${\bf{\Psi}}$ = (**W***_L_*)^T^, *L*-level SE can be directly integrated into the memCS framework without extra hardware overhead. Here, ${\mathrm{\bf W}}_i^\alpha ,\ \alpha $∈{+, −} represent the low-pass (+) and high-pass (−) filter matrices at level *i*, respectively. Each ${\mathrm{\bf W}}_i^\alpha $ consists of *N*/2*^i^* blocks of ${\mathrm{\bf w}}_i^\alpha $, where each ${\mathrm{\bf w}}_i^\alpha $ represents a unit vector with length 2*^i^* that extracts average or differential information of the signal at different scales, as shown in Equations (9) and (10).


(8) \begin{eqnarray*}
{\mathrm{\bf h}} = {{\mathrm{\bf W}}}_L \cdot \ {\mathrm{\bf x}} = \left( {\begin{array}{@{}*{1}{l}@{}} {{\mathrm{\bf W}}_L^ + }\\ {{\mathrm{\bf W}}_L^ - }\\ {{\mathrm{\bf W}}_{L - 1}^ - }\\ \vdots \\ {{\mathrm{\bf W}}_2^ - }\\ {{\mathrm{\bf W}}_1^ - } \end{array}} \right) \cdot {\mathrm{\bf x}}
\end{eqnarray*}

(9) \begin{eqnarray*}
{\mathrm{\bf W}}_i^\alpha = \left[ {\begin{array}{c@{\quad}c@{\quad}c@{\quad}c} {{\mathrm{\bf w}}_i^\alpha }&{}&{}&{}\\ {}&{{\mathrm{\bf w}}_i^\alpha }&{}&{}\\ {}&{}& \ddots &{}\\ {}&{}&{}&{{\mathrm{\bf w}}_i^\alpha } \end{array}} \right]
\end{eqnarray*}

(10) \begin{eqnarray*}
{\mathrm{\bf w}}_i^\alpha = \left[ {\underbrace {1,\ 1,\ \ldots ,\ 1}_{{2}^{i - 1}},\ \underbrace {\alpha 1,\ \alpha 1,\ \ldots ,\ \alpha 1}_{{2}^{i - 1}}} \right] \cdot {\left( {\sqrt 2 } \right)}^{ - i}
\end{eqnarray*}

Figure 5b demonstrates the sparsity reducing effect of the SE strategy. Compared to the scenario without SE, i.e. using a discrete cosine transform (DCT) matrix, the SE strategy leads to a dramatic decrease in sparsity from 166 to 22, significantly improving the convergence and reducing the implementation difficulty of CS reconstruction. Additionally, the level of SE should also be carefully selected. Theoretically, for a signal of length *N*, the available SE levels are *L* = 1, 2, …, ${L}_m$, where ${L}_m$ is the power of 2 in the prime factorization of *N*. For *N* = 256 in this work, the maximum SE level is set as *L_m_* = 8. Fig. 5c shows that memCS reconstruction accuracy exhibits significant improvement only as the SE level increases from *L* = 1–3, while variations are minimal within *L* = 4–8. Notably, when *L* > 5, as *L* increases, the complexity of the matrix element distribution of **W***_L_* would also increase, incorporating more intermediate memristor conductance states and thus resulting in higher sensitivity to memristor noise, which consequently leads to a degraded reconstruction accuracy. In subsequent experiments, *L* = 5 is selected to ensure effective sparsity enhancement and moderate element complexity of matrix **W***_L_*. The synergistic optimization effect of combining MMM and SE strategies is further validated through memCS-based image reconstruction experiments, as shown in Fig. 5d. When using SE alone, the accuracy improvement is only 1.16 dB compared to the baseline (without MMM and SE), whereas when adopting both SE and MMM, the reconstruction accuracy achieves a further improvement of 5.87 dB, demonstrating that these two strategies could synergistically achieve significant performance enhancement.

Similar to Fig. 4d and e, we further investigate the impact of mapping error and read noise with different SD levels on memCS reconstruction accuracy, as shown in Fig. 5e and f. Compared to the scenario without SE (red curves), employing SE (blue curves) leads to further accuracy optimization across the entire noise range, with approximately 1.5 dB improvement even at higher noise levels, demonstrating that SE can effectively improve the memCS reconstruction accuracy under varying intensities of memristor noise without introducing additional hardware overhead. Beyond the mapping error and read noise, Notes S5 and S6 further examine the efficacy of MMM and SE to suppress the adverse impact of other non-ideal characteristics (such as the IR drop effect and stuck-at fault), thus providing more evidence for the generalization ability and robustness of these two strategies.

## Comprehensive evaluation of memCS performance

**Figure 6.**
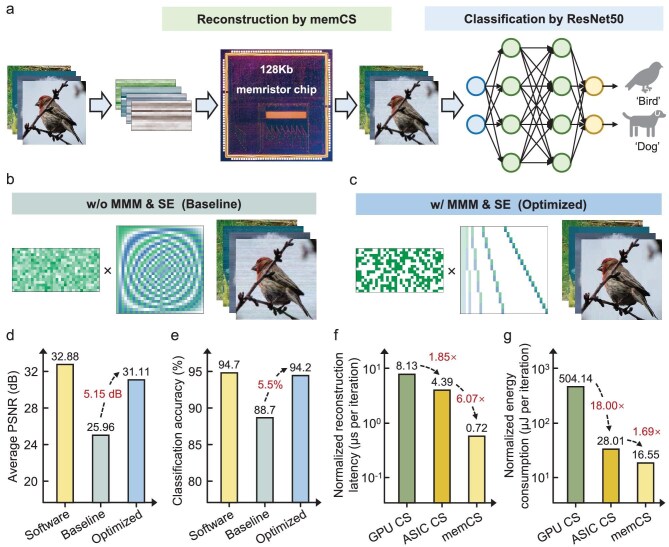
Performance evaluation of memCS. (a) Evaluation methodology for reconstruction accuracy, showing the workflow from image reconstruction to classification. (b and c) Composition of the weight matrix **A** and image reconstruction results by memCS with baseline (b) and optimized (c) configurations. w/o, without; w/, with. For each configuration, the same matrix **A** and **A**^T^ are used throughout the reconstruction process. (d and e) Comparison of reconstruction accuracy between software CS, baseline memCS and optimized memCS for average PSNR (d) and classification accuracy (e). (f and g) Comparison of normalized reconstruction latency (f) and energy consumption (g) between the GPU-based CS (GPU CS), ASIC-based CS (ASIC CS) and memCS.

Following the above co-optimization strategies, a comprehensive performance benchmark for memCS is conducted to demonstrate its practical performance for edge computing applications, including the reconstruction accuracy, latency and energy consumption. To evaluate the reconstruction accuracy, as illustrated in Fig. 6a, a collection of 1000 images spanning 50 categories from the ImageNet dataset is employed for comparative reconstruction experiments between software CS and memCS. Subsequently, a pre-trained ResNet50 convolutional neural network [44] is used to assess whether the key information in the reconstructed images is well preserved.

As illustrated in Fig. 6d and e, software CS achieves an average PSNR of 32.88 dB, while the baseline memCS (without MMM and SE) reaches 25.96 dB, with a 6.92 dB accuracy gap. In contrast, the optimized memCS (with MMM and SE) achieves an average PSNR of 31.11 dB, representing a 5.15 dB improvement over the baseline and significantly reducing the accuracy gap with software CS to only 1.77 dB. In addition, Fig. 6b and c depicts representative reconstructed images before and after optimization. Relative to the baseline, applying MMM and SE can effectively compress the traverse striping artifacts into minimal noise blocks, markedly enhancing the visual quality. Regarding the classification accuracy of reconstructed images, software CS and baseline memCS attain 94.7% and 88.7%, respectively, showing a 6.0% accuracy gap, while optimized memCS reaches 94.2%, improving by 5.5% over the baseline and reducing the gap with software CS to merely 0.5%, demonstrating that optimized memCS can effectively preserve the critical information of signals. Moreover, Note S4 presents additional examples of reconstructed signals by memCS from ImageNet and other datasets, demonstrating the broad applicability of the proposed optimization strategies across diverse datasets.

Furthermore, we evaluate the reconstruction latency and energy consumption of memCS, compared with both software and hardware counterparts. For the software CS, we employ the NVIDIA H100 GPU as the computing platform, which features high computing performance of 240 tera operations per second (TOPS) and 0.34 TOPS/W for 8-bit integer (INT8) operations [45]. For the hardware CS, we develop a dedicated digital circuit implementation as an application-specific integrated circuit (ASIC) for AMP algorithm reconstruction using 8-bit fixed-point arithmetic. Performance metrics for this implementation are calculated based on synthesis results of the 28 nm technology node by Design Compiler. For memCS, we construct a macro model based on our previous work [46–48] that integrates memristor arrays with peripheral circuits, with a Milk-V Meles single-board computer [49] for non-MVM computations. More details and discussion of the benchmark are provided in Notes S7–S10.

The evaluation results are illustrated in Fig. 6f and g. In terms of latency, the GPU CS and ASIC CS take 8.13 and 4.39 μs per iteration, respectively, whereas memCS significantly reduces this to 0.72 μs, achieving speedups of 11.22 and 6.07 times compared to the GPU CS and ASIC CS, respectively. In terms of energy consumption, the memCS consumes only 16.55 μJ per iteration, demonstrating 30.46 times and 1.69 times higher energy efficiency than those of the GPU CS and ASIC CS, respectively. In addition, the detailed performance analysis shows that for GPU CS, the latency and energy consumption of data transmission reach 7.76 μs/iteration and 392.31 μJ/iteration, which account for 95.43% and 77.82% of the total values. For ASIC CS, these values are 1.35 μs/iteration (30.77%) and 27.69 μJ/iteration (98.86%). In contrast, for memCS, with its CIM paradigm, they are significantly reduced to 56.09 ns/iteration (7.75%) and 1.28 μJ/iteration (7.74%). These results underscore the remarkable advantages of memCS in both speed and energy efficiency, highlighting its potential for power-limited edge computing applications. Meanwhile, extended experiments in Note S10 show that as the signal size increases, the performance advantages of memCS become increasingly pronounced, indicating strong scalability in larger-scale deployments.

## CONCLUSIONS

In this work, we present a memCS that leverages a fully integrated 128 Kb memristor chip to efficiently implement the AMP algorithm for iterative signal reconstruction. Through comprehensive experimental validations, we demonstrate the efficacy of memCS for the reconstruction of both 1D sparse signals and 2D natural images. To address the critical challenge of error accumulation in memristor-based iterative computations, we conduct a comprehensive analysis of memristor non-ideal characteristics and their impact on algorithmic performance, based on which we propose a hardware–software co-optimization framework for memCS that integrates the MMM and SE strategies. Experimental results demonstrate that these strategies significantly enhance the memCS’s robustness and improve the reconstruction accuracy. Consequently, the optimized memCS demonstrates remarkable performance, achieving a near-software reconstruction accuracy with an average PSNR of 31.11 dB for image reconstruction and a high accuracy of 94.2% for image classification on the ImageNet dataset. Furthermore, our performance evaluation reveals significant advantages over state-of-the-art CMOS implementations, with memCS operating 11.22 times faster and being 30.46 times more energy efficient compared to GPU-based CS. This work indicates that our memCS could achieve high reconstruction fidelity with prominent advantages in energy efficiency and computing speed through hardware–software co-optimization, thereby establishing a viable pathway toward energy-efficient CS systems for the edge scenarios with stringent power budgets. Future research may focus on architectural refinements, robustness enhancement techniques and integration into broader machine learning frameworks to further expand the application scope of memristor-based computing paradigms for signal processing applications.

## Supplementary Material

nwaf499_Supplemental_File

## Data Availability

The source data and code have been deposited in GitHub (https://github.com/Tsinghua-LEMON-Lab/memCS). Additional codes of this study are available from the corresponding authors upon reasonable request.
