# Supplementary material for: A memristor-based energy-efficient compressed sensing accelerator with hardware–software co-optimization for edge computing
Source: Natl Sci Rev. 2025 Nov 13;13(1):nwaf499. doi: 10.1093/nsr/nwaf499 (PMC12798729; doi:10.1093/nsr/nwaf499)
Supplement: nwaf499_Supplemental_File [file nwaf499_supplemental_file.pdf]

Supplementary Material for

A memristor-based energy-efficient compressed sensing accelerator with hardware-software co-optimization for edge computing

Yunrui Jiao[1,†], Han Zhao[1,†], Jianshi Tang[1*], Yanze Zhou[1], Ruofei Hu[1], Haochen Jiang[1], Xingchu Li[1], Jingyuan Huang[1], Biao Sun[2], Wen Sun[1], Bin Gao[1], He Qian[1] and Huaqiang Wu[1*]

[1]School of Integrated Circuits, Beijing Advanced Innovation Center for Integrated Circuits, BNRist, Tsinghua University, Beijing 100084, China

[2]School of Electrical Engineering and Automation, Tianjin University, Tianjin 300072, China

*E-mail: jtang@tsinghua.edu.cn; wuhq@tsinghua.edu.cn

[†]These authors contributed equally.

Table of contents:

Note 1: Programming procedures of the memristor array and additional test results

Fig. S1 Photographs of the test board of the 128Kb memristor chip.

Fig. S2 Standard deviation values (STD) of read noise across 10 conductance levels.

Note 2: Extended analysis of measurement matrix modification (MMM) strategy

Fig. S3 Sensitivity analysis of parameters α and β in the MMM strategy.

Note 3: Extended analysis of sparsity enhancement (SE) strategy

Fig. S4 Optimization effects of the SE strategy in signal reconstruction.

Note 4: More results of memCS reconstruction

Fig. S5 More examples of reconstructed images from the ImageNet dataset.

Fig. S6 More examples of reconstructed images from other datasets.

Fig. S7 More examples of reconstructed signals from the UCR Time Series Classification Archive.

Note 5: Analysis of relaxation effect and stuck-at fault on memCS

Fig. S8 Impact of the relaxation effect and stuck-at fault on memCS reconstruction.

Note 6: Analysis of IR drop effects on memCS and compensation strategy

Fig. S9 Parasitic resistance model of the memristor array.

Table. S1 The sheet resistance of interconnects on different metal layers in the layout of 128 Kb memristor chip.

Table. S2 Parasitic resistance values of the 128Kb memristor chip.

Fig. S10 The distortion of conductance matrix induced by IR drop.

Fig. S11 Signal reconstruction results during the iteration of IR drop compensation.

Table. S3 Parasitic resistance settings for 4 corners.

Fig. S12 Convergence of IR-drop compensation across 4 corners.

Note 7: Performance benchmark of GPU-based CS

Table. S4 Configuration table for GPU-based CS.

Table. S5 Breakdown of latency and energy consumption for GPU-based CS per iteration.

Note 8: Performance benchmark of ASIC-based CS

Table. S6 Configuration table for ASIC-based CS.

Table. S7 Breakdown of latency and energy consumption for ASIC-based CS per iteration.

Note 9: Performance benchmark of memCS

Fig. S13. Macro model of the memristor chips in memCS.

Table. S8 Benchmark details of the memristor chips in memCS.

Table. S9 Configuration table for Milk-V Meles SBC.

Table. S10 Configuration table for PCIe 5.0.

Table. S11 Breakdown of latency and energy consumption for memCS per iteration.

Note 10: Further discussion on performance benchmark

Fig. S14 Performance comparison between GPU-based CS and memCS across different input signal sizes (*N*).

Note 1: Programming procedures of the memristor array and additional test results

A memristor device typically starts at a low conductance and requires a high forward voltage to initiate the analog switching characteristics (i.e., the FORMING process). During the operation, when a forward voltage pulse is applied across the electrodes, oxygen ions in HfO_2 resistive-switching layer are driven to migrate, while oxygen vacancies remain localized and accumulate to form conductive filaments, thereby increasing the device conductance (i.e., the SET process). Conversely, a reverse voltage pulse applied across the electrodes induces the oxygen vacancies to redistribute or become trapped within HfO_2, rupturing the conductive filaments and reducing the device conductance (i.e. the RESET process). Additionally, to read out the memristor conductance (i.e., the READ process), a small voltage pulse (the amplitude is V_{read}) is applied across the electrodes, yielding a read current I_{read} that reflects the magnitude of the conductance $G = I_{read} / V_{read}$.

In this experiment, the pre-computed weight matrices $\mathbf{A}$ and $\mathbf{A}^T$ are first transformed to target conductance matrices via a quasi-analog mapping strategy [1], and then programmed into the memristor array using a write-verify scheme [2, 3]. Specifically, during programming, iterative SET and RESET operations are applied to selected cells to progressively approach the target conductance. If the conductance falls within a predefined error margin, the cell is marked as passing; otherwise, the write-verify cycle is repeated. The typical values of the SET, RESET, and READ voltage are 1.4 V, 1.5 V, and 0.2 V, respectively, with a pulse width of 200 μs. The error margin is set to ±1 μS (equivalently ±0.2 μA in the current). Meanwhile, a 200 μA compliance current is set to prevent excessive current from damaging the analog switching characteristics.

Based on the read currents measured after programming, we estimate the typical values of the memristor's mapping error and read noise. Figs. 2f and 2h show that both types of deviation approximately follow a Gaussian distribution. As a supplement, Figs. S2 present the standard deviation values (STD) of read noise across 10 conductance levels, revealing higher STD at intermediate conductance states and lower STD at low and high conductance states.

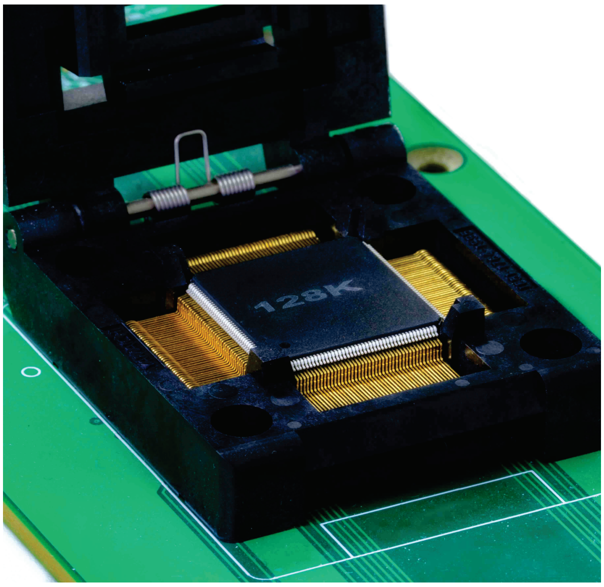

Fig. S1 Photographs of the test board of the 128Kb memristor chip.

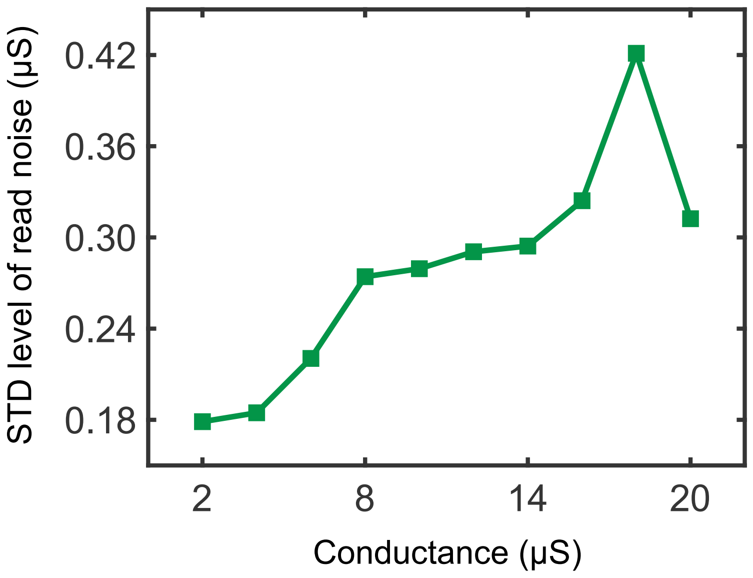

Fig. S2 Standard deviation values (STD) of read noise across 10 conductance levels. From 100 memristors at each conductance level and each device is read for 100 cycles.

Note 2: Extended analysis of measurement matrix modification (MMM) strategy

The measurement matrix modification (MMM) strategy aims to match the mean and standard deviation of the matrix entries before and after quantization, thereby reducing reliance on unstable intermediate conductance states while preserving the restricted isometry property of the measurement matrix. This enables the robustness of compressed sensing (CS) reconstruction against memristor noise. For a matrix **A**, if the inequality in Eq. (S1) holds for any *k*-sparse vector x (i.e., $||x||_0 \leq k$), then **A** is said to satisfy the *k*-order restricted isometry property. In CS, **A** equals the product of the measurement matrix **Φ** and the sparse transform matrix **Ψ**. Since common orthogonal transforms (e.g., DCT, DWT) generally preserve vector norms (i.e., $||\boldsymbol{\Psi}x||_2 = ||x||_2$ in the transform domain), it suffices for the measurement matrix **Φ** to satisfy the property.

$$(1-\delta_k)||x||_2^2 \leq ||\mathbf{A}x||_2^2 \leq (1+\delta_k)||x||_2^2 \tag{S1}$$

For an $M \times N$ Gaussian random matrix **Φ** whose entries are i.i.d. $\mathcal{N}(0, 1/M)$, according to ref. [4], if the number of measurements satisfies $M \geq C \cdot k \cdot \lg(N/k)$, then **Φ** satisfies the *k*-order restricted isometry property with overwhelmingly high probability. After quantization, the weights of $Q_{MMM}(\boldsymbol{\Phi})$ exhibit deviations in both mean and standard deviation. After applying the linear calibration in Eqs. (S2)-(S4), $\boldsymbol{\Phi}_{MMM}$ can be guaranteed to preserve the same mean and standard deviation as **Φ**.

$$\boldsymbol{\Phi}_{MMM} = \beta \cdot Q_{MMM}(\boldsymbol{\Phi}) + \alpha \tag{S2}$$

$$\beta = \frac{\sigma(\boldsymbol{\Phi})}{\sigma\left(Q_{MMM}(\boldsymbol{\Phi})\right)} = \frac{1}{M\sigma\left(Q_{MMM}(\boldsymbol{\Phi})\right)} \tag{S3}$$

$$\alpha = \mu(\boldsymbol{\Phi}) - \beta \cdot \mu\left(Q_{MMM}(\boldsymbol{\Phi})\right) = -\frac{\mu\left(Q_{MMM}(\boldsymbol{\Phi})\right)}{M\sigma\left(Q_{MMM}(\boldsymbol{\Phi})\right)} \tag{S4}$$

Assuming **Φ** satisfies Eq. (S1), then for $\boldsymbol{\Phi}_{MMM}$ and an arbitrarily vector x, we calculate the mathematical expectation of $||\boldsymbol{\Phi}_{MMM}x||_2^2$:

$$E(||\boldsymbol{\Phi}_{MMM}x||_2^2) = E\left(\sum_{i=1}^{M}\left(\sum_{j=1}^{N}\boldsymbol{\Phi}_{MMM,ij}\,x_j\right)^2\right) = \sum_{i=1}^{M} E\left(\sum_{j=1}^{N}\boldsymbol{\Phi}_{MMM,ij}\,x_j\right)^2 \tag{S5}$$

We denote:

$$Y_i = \sum_{j=1}^{N}\boldsymbol{\Phi}_{MMM,ij}\,x_j \tag{S6}$$

Then Eq. (S5) can be turned into: ($Var(\cdot)$ refers to the variation operator)

$$E(||\boldsymbol{\Phi}_{MMM}x||_2^2) = \sum_{i=1}^{M} E(Y_i)^2 = \sum_{i=1}^{M}\left(E(Y_i)\right)^2 + \sum_{i=1}^{M} Var(Y_i) \tag{S7}$$

The two terms can be derived respectively: (as $E\left(\mathbf{\Phi}_{\mathrm{MMM},ij}\right) = E(\mathbf{\Phi}) = 0$, $Var\left(\mathbf{\Phi}_{\mathrm{MMM},ij}\right) = Var\left(\mathbf{\Phi}_{ij}\right) = 1/M$)

$$E(Y_i) = E\left(\sum_{j=1}^{N} \mathbf{\Phi}_{\mathrm{MMM},ij}\, \mathrm{x}_j\right) = \sum_{j=1}^{N} E\left(\mathbf{\Phi}_{\mathrm{MMM},ij}\right) \mathrm{x}_j = \sum_{j=1}^{N} 0 \cdot \mathrm{x}_j = 0 \tag{S8}$$

$$Var(Y_i) = Var\left(\sum_{j=1}^{N} \mathbf{\Phi}_{\mathrm{MMM},ij}\, \mathrm{x}_j\right) = \sum_{j=1}^{N} Var\left(\mathbf{\Phi}_{\mathrm{MMM},ij}\right) \mathrm{x}_j^2 = \sum_{j=1}^{N} \frac{1}{M} \mathrm{x}_j^2 = \frac{\|\mathrm{x}\|_2^2}{M} \tag{S9}$$

Subsequently, the mathematical expression in Eq. (S7) can be calculated:

$$E(\|\mathbf{\Phi}_{\mathrm{MMM}}\mathrm{x}\|_2^2) = \sum_{i=1}^{M} (0)^2 + \sum_{i=1}^{M} \frac{\|\mathrm{x}\|_2^2}{M} = \|\mathrm{x}\|_2^2 \tag{S10}$$

This result demonstrates that under the linear calibration of Eqs. (S2)-(S4), the matrix $\mathbf{\Phi}_{\mathrm{MMM}}$ preserves the Euclidean length of vectors in the sense of mathematical expectation. Specifically, the matrix elements $\mathbf{\Phi}_{\mathrm{MMM,\ ij}}$ are obtained by quantizing, scaling, and translating from the original Gaussian variables. Since the quantization operation maps continuous values to a finite set of discrete values, $\mathbf{\Phi}_{\mathrm{MMM,\ ij}}$ is a bounded random variable. By the Concentration Inequalities in ref. [4], this implies that the random quantity $\|\mathbf{\Phi}_{\mathrm{MMM}}\mathrm{x}\|_2^2$ will fall within a small neighborhood of its expected value $\|\mathrm{x}\|_2^2$ with extremely high probability, which satisfies the definition of restricted isometry property in Eq. (S1). This indicates that matrix $\mathbf{\Phi}_{\mathrm{MMM}}$ processed by the MMM strategy is a high-quality measurement matrix. The experimental results presented in the main text also provide strong empirical support for this argument.

Furthermore, we investigate the parameter sensitivity of α and β in the MMM strategy. When α and β deviate from the target values α_0 and β_0 in Eqs. (S3)-(S4) (i.e., α =(1+e_α)·α_0, β =(1+e_β)· β_0, e_α, e_β are the relative error of α and β). The results are shown in Fig. S3. Within a tolerance corresponding to a twofold accuracy drop (-3 dB), α and β exhibit parameter robustness of ±68% and ±14%, respectively, indicating that the parameters have considerable tolerance for relative error.

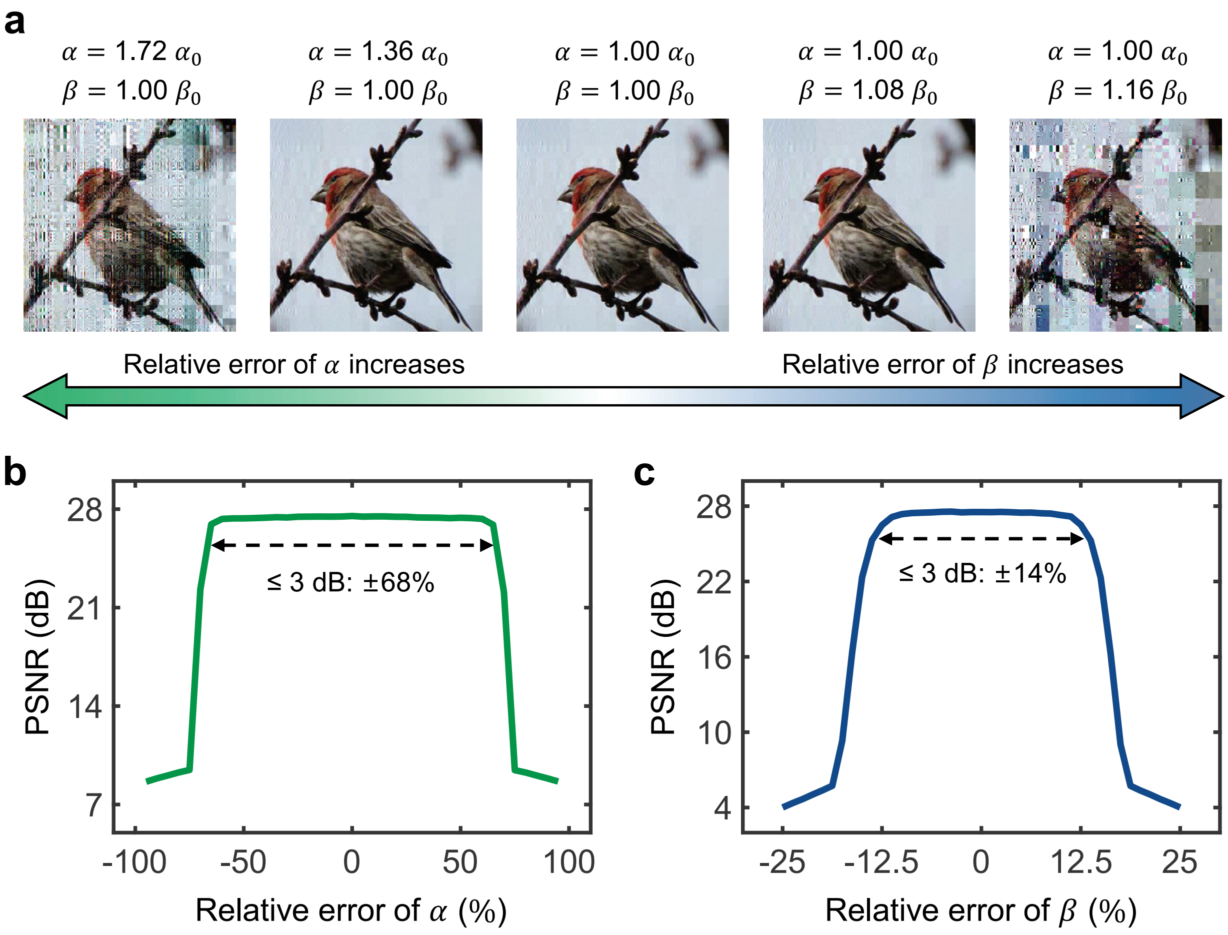

Fig. S3 Sensitivity analysis of parameters α and β in the MMM strategy. (a) Examples of reconstructed images with deviated parameters α and β. (b)-(c) Reconstruction accuracy with various relative error of parameter α or β, remains stable within a certain range and drops rapidly once this range is exceeded.

Note 3: Extended analysis of sparsity enhancement (SE) strategy

The sparsity enhancement (SE) strategy based on multi-level Haar discrete wavelet transform (DWT) reflects a trade-off between algorithmic performance and memristor compatibility. Specifically, the multi-level Haar DWT employs basis functions composed of piecewise-constant square waves. Consequently, it is exceptionally effective at representing signals characterized by piecewise regularities and sharp local transitions. These features are fundamental to a wide range of data, especially the edges and contours that define images, as well as transient events in time-series data like certain bioelectrical signals [5, 6]. This inherent suitability allows Haar bases to efficiently capture multi-scale characteristics by adjusting the number of decomposition levels, effectively enhanced the sparsity, and thereby enable high-accuracy CS reconstruction, as illustrated in Fig. S4. According to [7], the applicability of the SE strategy depends on how well the transform basis matches the intrinsic structure of the signal. For example, SE may fail to significantly enhance sparsity for smooth signals, which in turn limits improvements in reconstruction accuracy.

In addition, Eqs. (8)-(10) in the main text show that SE can be directly integrated into the memCS framework without any extra hardware overhead. Moreover, in the memCS implementation of CS reconstruction, both the MMM and SE strategies are executed during the preprocessing stage (i.e., step ‘Get initial matrices’ in Fig. 1d of the main text). Since these strategies only modify the weight values of the matrix without altering its dimensions, the computational complexity per iteration remains unchanged. Thus, their energy consumption and latency are negligible in the evaluation.

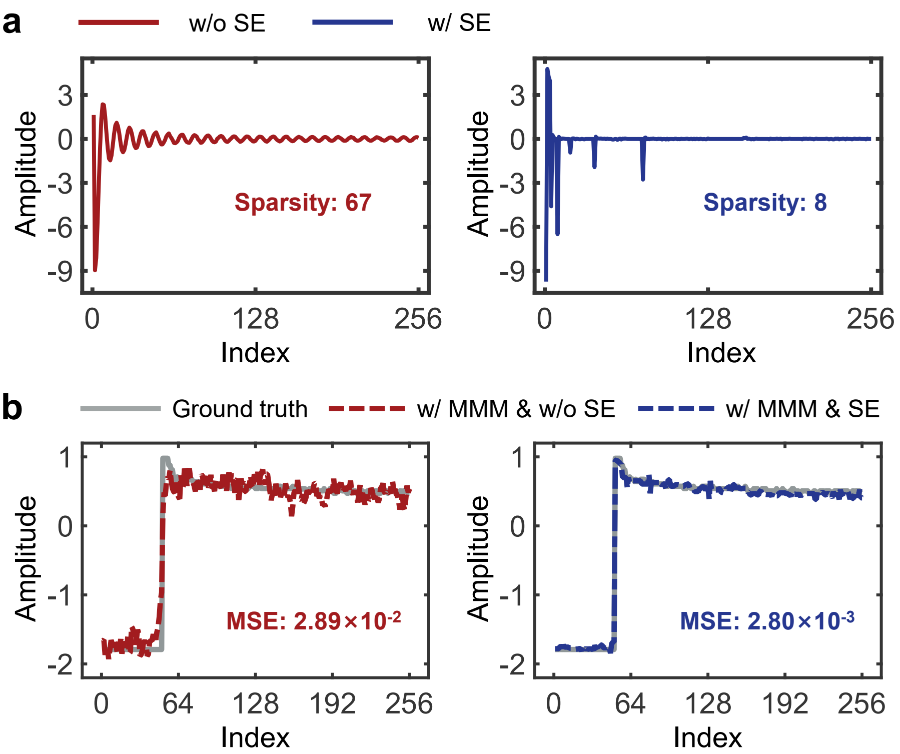

Fig. S4 Optimization effects of the SE strategy in signal reconstruction. The original signal belongs to the training set of Freezer Regular Train (Sensor signal) dataset in the UCR Time Series Classification Archive [8]. (a) Sparsity optimization. (b) Reconstruction fidelity optimization. w/o: without; w/: with.

Note 4: More results of memCS reconstruction

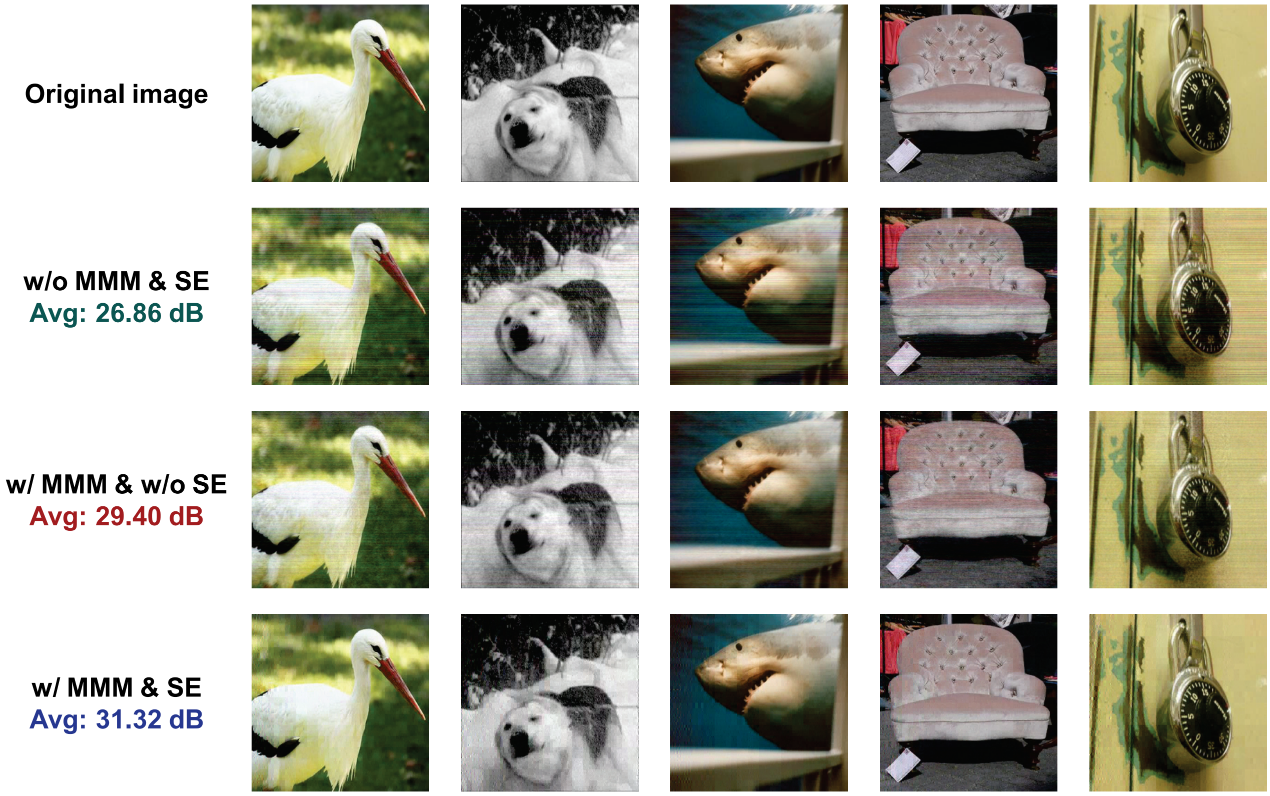

Fig. S5 More examples of reconstructed images from the ImageNet dataset. Both the visual quality and PSNR of reconstructed images increases when employing the MMM and SE optimization strategies. w/o: without, w/: with.

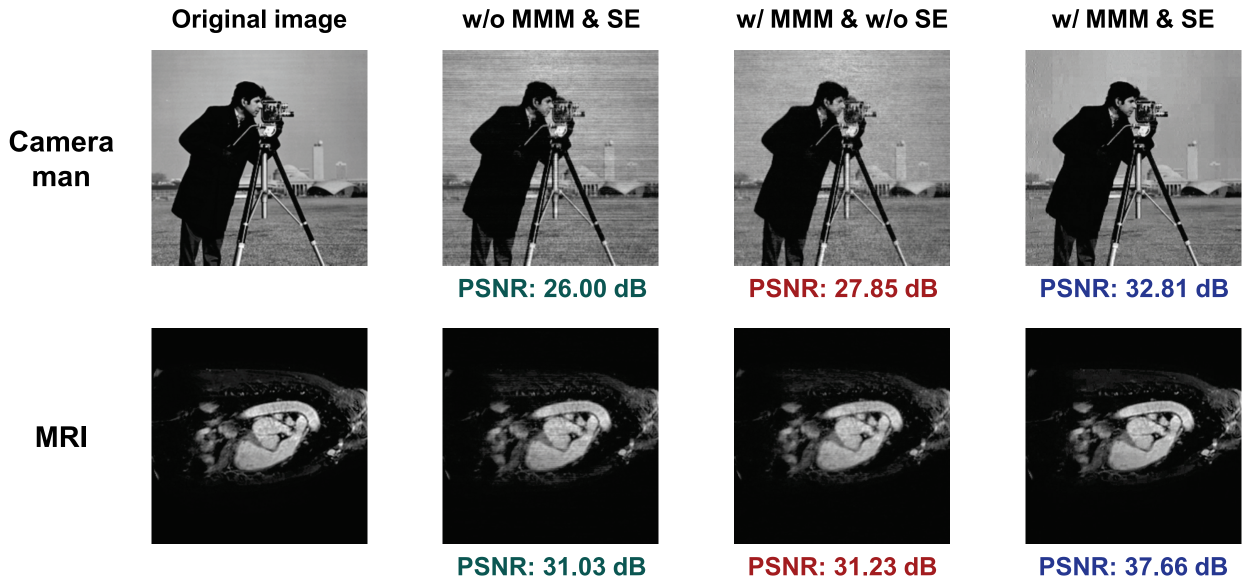

Fig. S6 More examples of reconstructed images from other datasets. Cameraman: the classic test image widely used in image processing research. MRI (Magnetic Resonance Imaging): medical imaging dataset comprising multi-contrast magnetic resonance images. In the memCS reconstruction, the input images are sliced and resized to 256×256. w/o: without, w/: with.

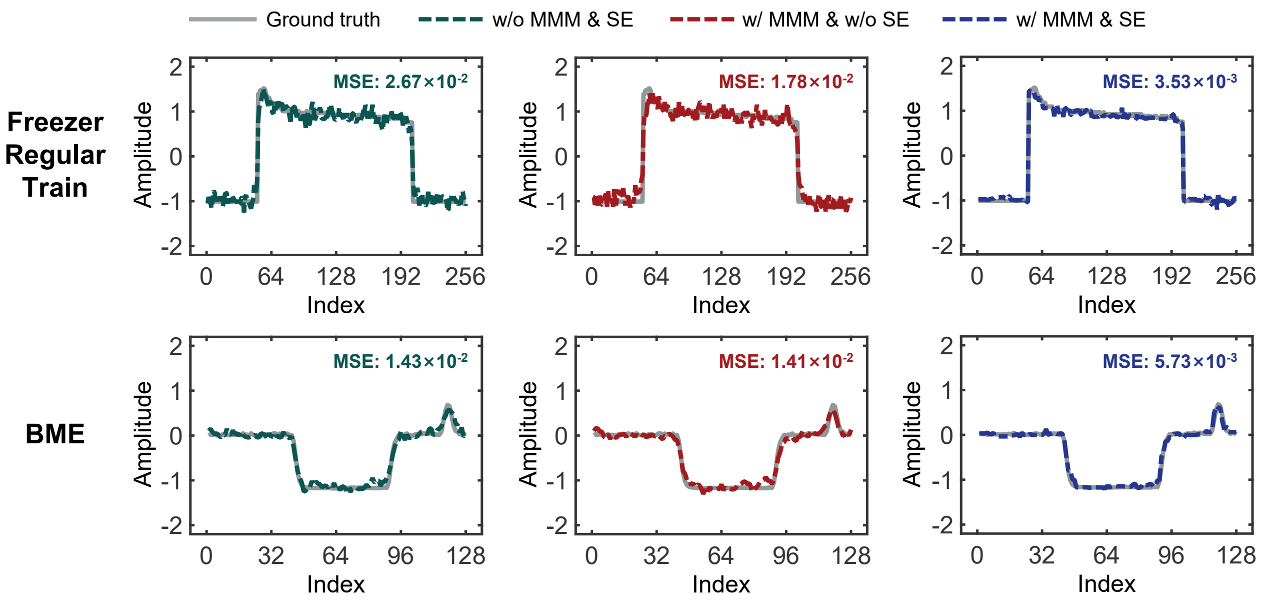

Fig. S7 More examples of reconstructed signals from the UCR Time Series Classification Archive [8]. The original signals come from two training set categories: Free Regular Train (Sensor signal) and BME (Simulated signal). The reconstruction results show that our optimization strategy improves the accuracy by 7.6× and 2.5×, respectively. w/o: without; w/: with.

Note 5: Analysis of relaxation effect and stuck-at fault on memCS

In memristor chips, the relaxation effect refers to the spontaneous drift of a device's stored conductance value away from its initial value. The relative magnitude of this effect reflects the data retention capability of the memristor device over time. Following ref. [9], we model the conductance under the influence of relaxation as a Gaussian random variable with a mean equal to the post-programming conductance and a standard deviation of σ_R. Then we simulated the memCS reconstruction accuracy as a function of σ_R, comparing the performance before and after optimization with the MMM and SE strategies. As shown in Fig. S8a, the results indicate that the MMM and SE strategies consistently deliver an improvement of over 6 dB and are resistant against the relaxation effect.

A stuck-at fault indicates that a memory cell becomes fixed at a high conductance (stuck-at-ON) or low conductance (stuck-at-OFF) state, rendering it unable to be programmed to a target conductance value. The incidence of such faults is a key metric for device endurance after numerous programming cycles. Assuming a given fault rate where stuck-at-ON and stuck-at-OFF faults are equally probable (50% each), we evaluate the memCS reconstruction accuracy against the fault rate both with and without the MMM and SE optimizations. The results in Fig. S8b demonstrate that for fault rates up to 1%, the MMM and SE strategies yield a performance enhancement of at least 3 dB. Notably, when the fault rate is below 0.1% (which is consistent with our experimental measured results, i.e., our yield is >99.9% [10]), there is negligible degradation in reconstruction accuracy.

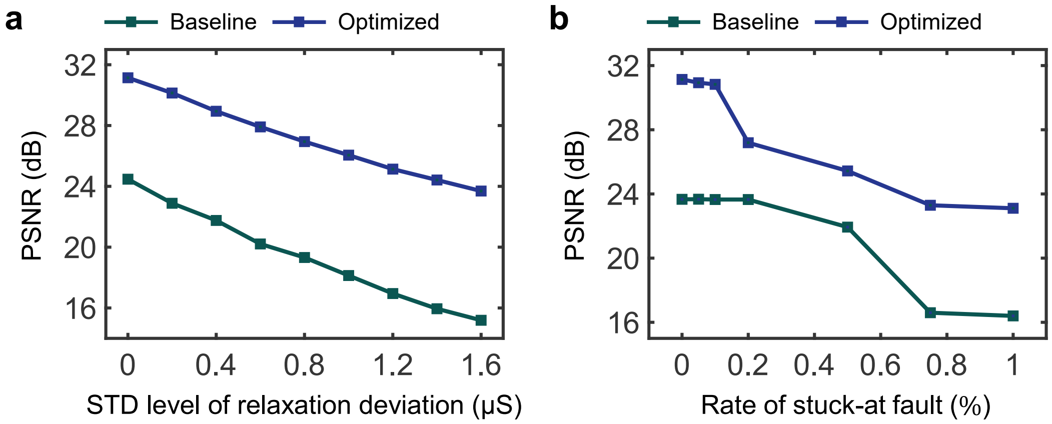

Fig. S8 Impact of the relaxation effect and stuck-at fault on memCS reconstruction. (a) Reconstruction accuracy on various STD levels of relaxation deviation. The effect of relaxation on conductance values is modelled as a Gaussian random variable with the post-programming conductance value as its mean. (b) Reconstruction accuracy on various percentage of stuck-at faults. Fault modes stuck-at-ON and stuck-at-OFF are each assigned a 50% probability in the simulation. Baseline: without MMM and SE strategies; Optimized: with MMM and SE strategies.

Note 6: Analysis of IR drop effects on memCS and compensation strategy

Beyond the device-level non-idealities discussed above, array-level parasitic effects such as IR drop also affect the computing accuracy of memristor-based systems and limit their scalability to larger array dimensions. In this Note, we examine how IR drop influences memCS reconstruction fidelity using a 128 Kb chip (the array size is 128×1024, and it is fabricated in a 130 nm process).

To assess the effect, we employ an IR-drop compact model that has been calibrated against measurements, as illustrated in Fig. S9. The model is established by mathematical induction and matches the experimental data closely. It includes four categories of parasitic resistance: the wire resistance of BLs (R_{bl}), wire resistance of SLs (R_{sl}), access resistance of BLs (R_{bl_acc}), and access resistance of SLs (R_{sl_acc}). Based on the 130 nm Process Design Kit (PDK) and the chip layout, we extracted the parasitic resistances for the 128 Kb chip, with the results summarized in Tables. S1 and S2. The SL access line is the longest segment (261.8 μm), and the typical value of R_{sl_acc} reaches 59.03 Ω, which dominates the IR-drop-induced performance loss. The BL access lines appear in four different lengths (19.8 μm, 20.4 μm, 80.4 μm and 83.4 μm) on the layout. To maintain a conservative margin, we use the longest 3.40 μm for the parasitic estimation, yielding a R_{bl_acc} of 22.55 Ω. In addition, the typical value of R_{bl} and R_{sl} are 0.09 Ω and 0.74 Ω, respectively.

Regarding the impact of IR drop, the compact model in ref. [3] indicates that it reduces the voltage across the electrodes of a memristor device, which in turn lowers the output current for a given array input voltage. This effect can be modeled as a distortion of the conductance matrix, as shown in Eqs. (S11)-(S12). Here, $\mathbf{G}_{ideal}$ represents the target conductance matrix, while $\mathbf{G}_{IR}$ is the distorted matrix resulting from the IR drop effect. $\mathbf{D}_R$ and $\mathbf{D}_C$ are diagonal matrices derived from the compact model and parasitic resistance values, where the diagonal elements represent the distortion factors. Fig. S10 illustrates the distortion of the conductance matrix (mapped from the weight matrix **A** after applying the MMM and SE optimization strategies) under the influence of IR drop. The conductance values of all elements in $\mathbf{G}_{ideal}$ are diminished to varying degrees, with a maximum reduction of 5.31 μS. This causes an amplitude deviation in the reconstructed signal (Fig. S11b) and degrades the accuracy by approximately an order of magnitude.

$$\mathbf{G}_{\mathrm{IR}} = \mathbf{D}_{\mathrm{R}} \cdot \mathbf{G}_{\mathrm{ideal}} \cdot \mathbf{D}_{\mathrm{C}} \tag{S11}$$

$$(\mathbf{D}_{\mathrm{R}}, \mathbf{D}_{\mathrm{C}}) = \mathrm{IR\_drop\_model}\,(\mathbf{G}_{\mathrm{ideal}}, R_{\mathrm{bl}}, R_{\mathrm{sl}}, R_{\mathrm{bl\_acc}}, R_{\mathrm{sl\_acc}}) \tag{S12}$$

To mitigate the impact of IR drop, we employ the compensation strategy proposed in ref. [11] to improve the reconstruction accuracy. The flowchart of the compensation algorithm is shown in Fig. S11a. First, a portion of the weights is fixed based on a set threshold. For the remaining weights that require updating, an iterative process is initiated. In each iteration, the distortion matrix is first calculated from the current conductance matrix $\mathbf{G}_i$ using the compact model. Subsequently, a Distortion Factor Matrix (DFM) is computed via element-wise division, and this DFM is used to calculate the updated conductance matrix $\mathbf{G}_{i+1}$. When $\mathbf{G}_{i+1}$ converges to $\mathbf{G}_i$, the updated matrix from this iteration, $\mathbf{G}_{i+1}$, is output as the final IR drop-compensated conductance matrix $\mathbf{G}_{\mathrm{comp}}$. In practical application, this strategy only needs to be executed once during the pre-processing stage (i.e., between the 'Calculate the weight matrix' and 'Map **A** and $\mathbf{A}^T$ on memristor arrays' steps in Fig. 1d of the main text). Therefore, the additional overhead is negligible.

In this experiment, we set the update threshold to 0. To enhance the convergence speed during the early stages, we also modified the DFM calculation, replacing the denominator $\mathbf{G}_i$ from ref. [11] with $\mathbf{G}_{\mathrm{ideal}}$. We then evaluated the memCS signal reconstruction at each step of the iteration, with the results depicted in Figs. S11b-11d. The figures clearly show the reconstructed signal's amplitude gradually recovering to the ground truth, yielding a substantial accuracy improvement in three iterations. Furthermore, we extended our analysis to three distinct corners, each defined by a dominant parasitic resistance (Table. S3). The convergence behavior of the mean squared error (MSE) for these corners is plotted in Fig. S12. The results reveal that R_{sl} exerts the most pronounced influence on reconstruction quality when parasitic resistances are held constant. Critically, the MSE for every corner stabilizes below 2.5×10^{-3} in 10 iterations, confirming the broad applicability of this IR drop compensation approach.

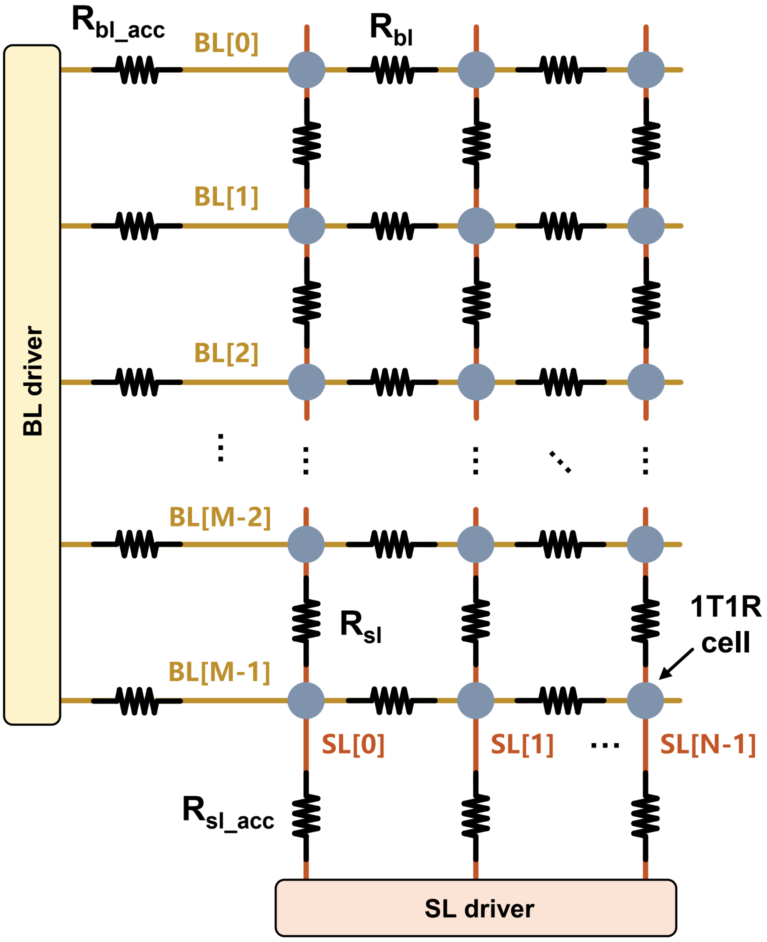

Fig. S9 Parasitic resistance model of the memristor array. Include R_{bl}, R_{sl}, R_{bl_acc}, and R_{sl_acc}.

**Metal layer**	**Sheet resistance (mΩ/sq)**
Metal 1	124
Metal 2	125
Metal 3	77
Metal 4	77
Metal 5 (Top Metal)	45

Table. S1 The sheet resistance of interconnects on different metal layers in the layout of 128 Kb memristor chip. Metal *i*: the *i*-th metal layer.

**Type**	**Metal layer**	**Width (µm)**	**Length (µm)**	**Typical Resistance (Ω)**
R_{bl} (Ω)	Metal 5	0.7	1.4	0.09
R_{sl} (Ω)	Metal 2,3	0.36	2.7	0.74
R_{bl_acc} (Ω)	Metal 2,3,4	0.26~0.36	19.8~83.4	22.55
R_{sl_acc} (Ω)	Metal 2,3,4	0.36~1	261.8	59.03

Table. S2 Parasitic resistance values of the 128Kb memristor chip. Metal *i*: the *i*-th metal layer.

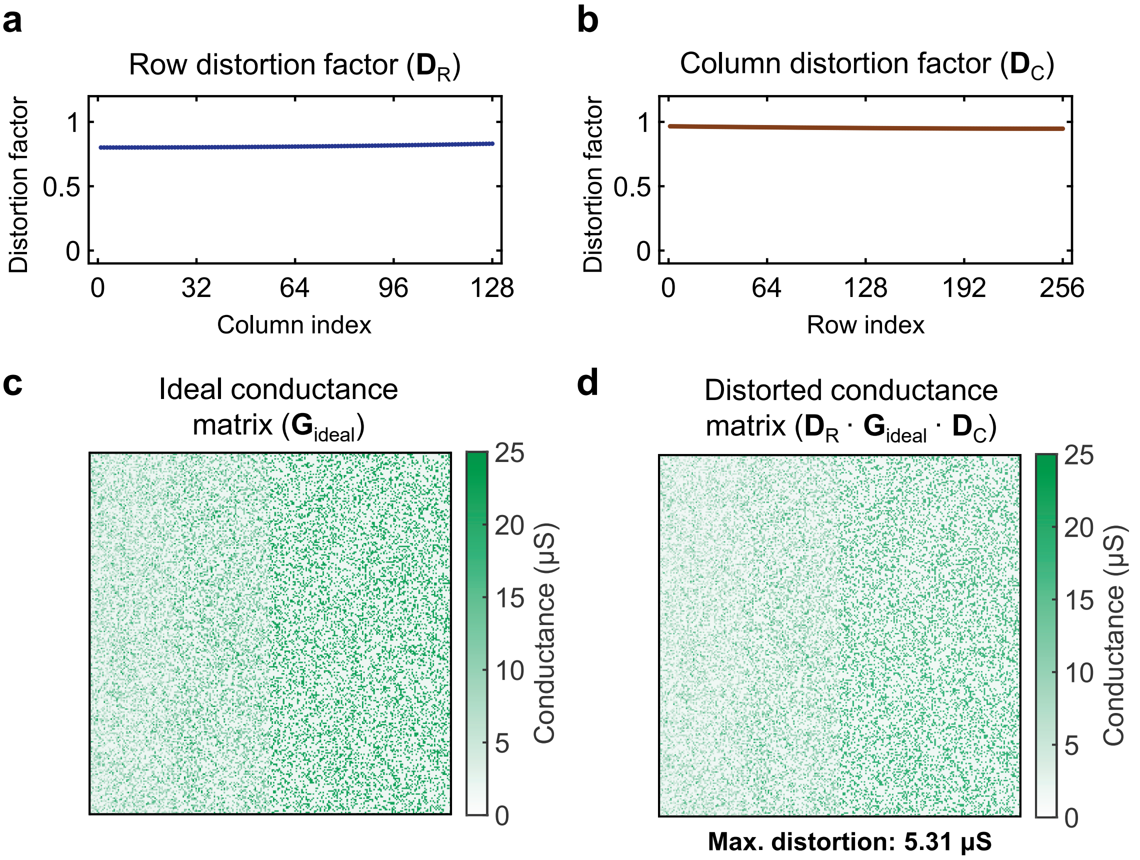

Fig. S10 The distortion of conductance matrix induced by IR drop. (a)-(b) Coefficients of the flattened diagonal distortion matrix $\mathbf{D}_R$ and $\mathbf{D}_C$ (i.e., distortion factor). (c) The ideal conductance matrix $\mathbf{G}_{ideal}$, (d) The conductance matrix $\mathbf{G}_{IR}$ effected by IR drop, with the max distortion value of 5.31 μS less than the $\mathbf{G}_{ideal}$.

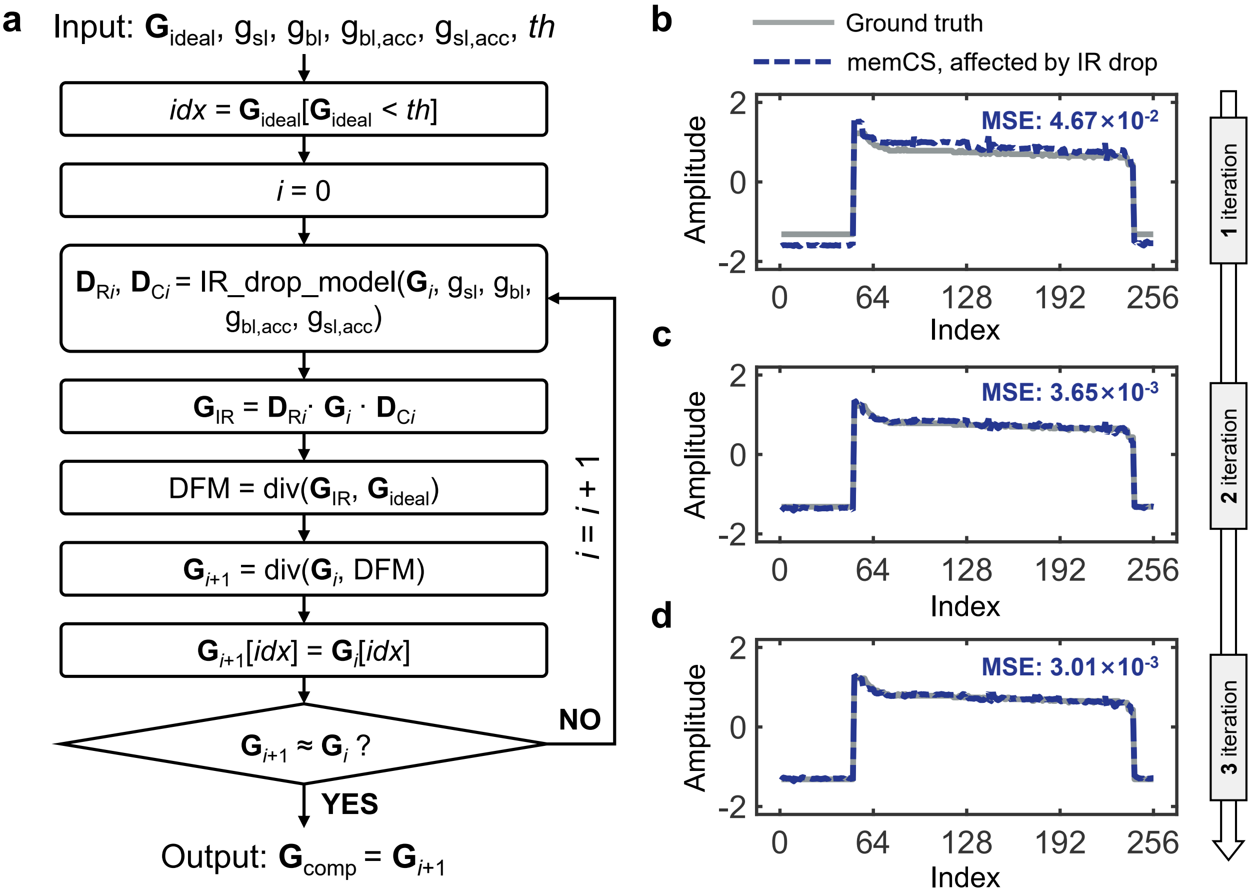

Fig. S11 Signal reconstruction results during the iteration of IR drop compensation. (a) Flowchart of the IR drop compensation algorithm. (b)-(d) Signal reconstruction results at the first three rounds of iteration, demonstrated significant accuracy improvement.

Type	Corner 1	Corner 2	Corner 3	128Kb memristor chip
R_{bl} (Ω)	**5.00**	0.50	0.50	0.09
R_{sl} (Ω)	0.50	**5.00**	0.50	0.74
R_{bl_acc} (Ω)	25.00	25.00	**100.00**	22.55
R_{sl_acc} (Ω)	25.00	25.00	25.00	**59.03**

Table. S3 Parasitic resistance settings for 4 corners. the values for the 128 Kb memristor chip are extracted from the PDK and the chip layout, while the other three corners are manually specified.

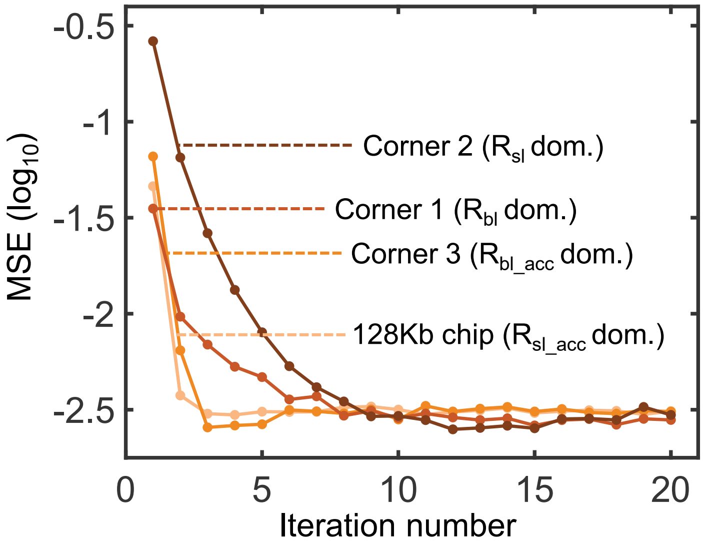

Fig. S12 Convergence of IR-drop compensation across 4 corners. The reconstruction accuracy converges for all corners after roughly 10 compensation iterations. dom., dominant.

Note 7: Performance benchmark of GPU-based CS

In the GPU-based CS implementation, we assume that both the MVM and non-MVM computations of each iteration in the reconstruction algorithm are executed on the NVIDIA H100 GPU [12]. The reading and storing of the matrix $\mathbf{A}$, $\mathbf{A}^T$, along with the intermediate computing results are all considered as interactions with GPU memory. The configuration parameters for the GPU-based CS are summarized in Table. S4. The computing workload and data transmission volume are derived from the algorithm flowchart in Fig. 1b of the main text.

Table. S5 presents the breakdown of reconstruction latency and energy consumption for a single iteration, showing that data transmission accounts for the majority of both metrics (95.43% for latency and 77.82% for energy consumption). It should be noted that when the process node scales from x nm to $n \cdot x$ nm, assuming the sheet resistance and supply voltage (V_{dd}) remain constant, the capacitance scales by a factor of n^2. Based on the RC time constant ($\tau \sim RC$) and capacitor's energy consumption ($E \sim CV^2$), the resulting normalization factor for both delay and energy is n^2.

Term	Value
GPU rated power	700 W
GPU energy efficiency	240 TOPS/W
Energy efficiency for data transmission	2 pJ/bit
Graphics memory bandwidth	3 TB/s
Process node	4 nm
Data type	INT8

Table. S4 Configuration table for GPU-based CS. As a conservative benchmark, the power consumption is assumed to be 350 W, representing half of the maximum value. Finally, both latency and energy consumption are normalized to the 130 nm process node.

**Reconstruction latency (ns/iteration)**			
**MVM**	**Non-MVM**	**Data transmission**	**Total**
368.10	3.24	7759.13	8130.47
**Energy consumption (μJ/iteration)**			
**MVM**	**Non-MVM**	**Data transmission**	**Total**
110.43	1.40	392.31	504.14

Table. S5 Breakdown of latency and energy consumption for GPU-based CS per iteration.

Note 8: Performance benchmark of ASIC-based CS

For the benchmark evaluation of ASIC-based CS, we have designed a digital ASIC chip based on 8-bit fixed-point arithmetic to implement the AMP algorithm flowchart depicted in Fig. 1b. The complete design was then synthesized with the Synopsys Design Compiler (DC). The synthesis report indicates that at a clock frequency of 125 MHz, all four non-MVM modules can complete one computation within a single clock cycle, whereas each of the two MVM modules requires 16 cycles to produce the output. The configuration parameters for the ASIC-based CS are provided in Table. S6. Table. S7 shows the detailed evaluation results. Due to the low energy efficiency of data transmission, it still remains the dominant contributor to energy consumption, accounting for 98.86% of the total.

Term	Value
Energy efficiency for data transmission	10 pJ/bit
Bandwidth for data transmission	256 GB/s
Process node	40 nm
Data type	INT8

Table. S6 Configuration table for ASIC-based CS. The metrics for the computation part are derived from the Design Compiler (DC) synthesis report, while the latency and energy consumption for data transmission are estimated using the parameters listed in this table. The energy efficiency and bandwidth for the memory interface are adopted from ref. [13-15]. Finally, both latency and energy consumption are normalized to the 130 nm process node for comparison.

**Reconstruction latency (ns/iteration)**			
**MVM**	**Non-MVM**	**Data transmission**	**Total**
2704.00	338.00	1352.00	4394.00
**Energy consumption (μJ/iteration)**			
**MVM**	**Non-MVM**	**Data transmission**	**Total**
0.31	0.005	27.69	28.01

Table. S7 Breakdown of latency and energy consumption for ASIC-based CS per iteration.

Note 9: Performance benchmark of memCS

For memCS, the macro model of the memristor chips is illustrated in Fig. S13. In MVM computation, the input voltage signals are applied to the BLs of the memristor array, where the matrix **A** or $\mathbf{A}^T$ is programmed. The output currents on SLs are selected by WL drivers and then read out by ADCs, representing the MVM results. The benchmark result for the memristor array is provided in Table. S8, and the details are shown as follows:

a) BL/WL/SL Drivers: The latency and energy consumption of the BL/WL/SL drivers are derived from post-layout simulations of a 128 Kb memristor chip. In terms of latency, the simulated read latencies for the BL, WL, and SL drivers of a 128×256 array are 5.05 ns, 5.20 ns, and 6.07 ns, respectively. Since each iteration involves two matrix-vector multiplication (MVM) steps, this combined latency is doubled, yielding a total driver latency of approximately 32.64 ns. As for energy consumption, the dynamic energy for activating 128 BLs, 256 WLs, and 256 SLs is 3.83 pJ, 9.36 pJ, and 3.84 pJ, respectively. When these values are extrapolated to a uniform configuration of 384 lines for each type, the total dynamic energy for the drivers is estimated to be 31.29 pJ.

b) Memristor array: The intrinsic latency of the memristor array itself is considered negligible compared to that of the peripheral circuits. This is justified by the rapid stabilization of the current on the SLs during a read operation. In terms of energy consumption, simulation results indicate that a single parallel read operation on the 128×256 array consumes 171.26 pJ. Accounting for the two MVM steps per iteration, the total energy consumption for the array is 342.52 pJ (171.26 pJ × 2).

c) ADC: The performance of the ADC module is benchmarked against the design presented in [16]. For latency, the referenced ADC features a sampling rate of 1 GS/s, which corresponds to a sampling period of 1 ns. As each iteration requires sampling the results of two separate MVM computations, the total latency contributed by the ADC is 2 ns. Regarding the energy consumption, The ADC has a reported energy efficiency of 3.8 pJ/S. To mitigate potential sampling errors and improve the signal-to-noise ratio (SNR), we assume an oversampling strategy where 1024 samples are acquired for each MVM result within one iteration. Consequently, the total energy consumption for the ADC is estimated to be 3.89 nJ.

d) Sample & Hold (S&H): The latency of the S&H circuit is considered negligible. This is justified because the high sampling rate of the ADC allows for the use of a small sampling capacitor, which in turn leads to a minimal RC time constant. In terms of energy consumption, we refer to the design in [10], which reports approximately 0.325 pJ per S&H operation. Consequently,

the total energy for the two operations required in each iteration is 0.65 pJ, representing a minor fraction of the overall system's energy budget.

e) Shift & Add (S&A): The overhead of the Shift-and-Add (S&A) module is estimated based on the data presented in [10]. The latency is primarily determined by the internal registers, which introduce a delay of one clock cycle (approximated as 5 ns) per operation. Thus, the two S&A operations required per iteration contribute a total of 10 ns to the latency. For energy consumption, the reference indicates that a block of 32 S&A units implemented in a 130 nm technology node consumes 16.26 pJ. By linearly scaling this to our architecture's requirement of total 384 units, the energy consumption is calculated to be 195.12 pJ.

Besides, non-MVM computing are performed on a Milk-V Meles single-board computer (SBC) equipped with a quad-core RISC-V 64GCV C910 processor [17]. In addition, PCI Express (PCIe) 5.0 protocol is selected for data transmission between the memristor chips and the SBC [18]. The configuration parameters for these parts are summarized in Table. S9 and Table. S10.

The evaluation results of memCS in Table. S11 indicate that, compared to ASIC-based CS, memCS leverages in-situ MVM to achieve more than an order-of-magnitude reduction in the latency and energy consumption associated with MVM computation and data transmission. However, due to the use of a mature 130 nm technology node, the overhead from the peripheral non-MVM computation is relatively pronounced compared to its GPU and ASIC counterparts, accounting for a substantial 86.09% and 92.23% of the total system overhead, respectively. This limitation, however, can be addressed in future work by migrating to a more advanced technology node.

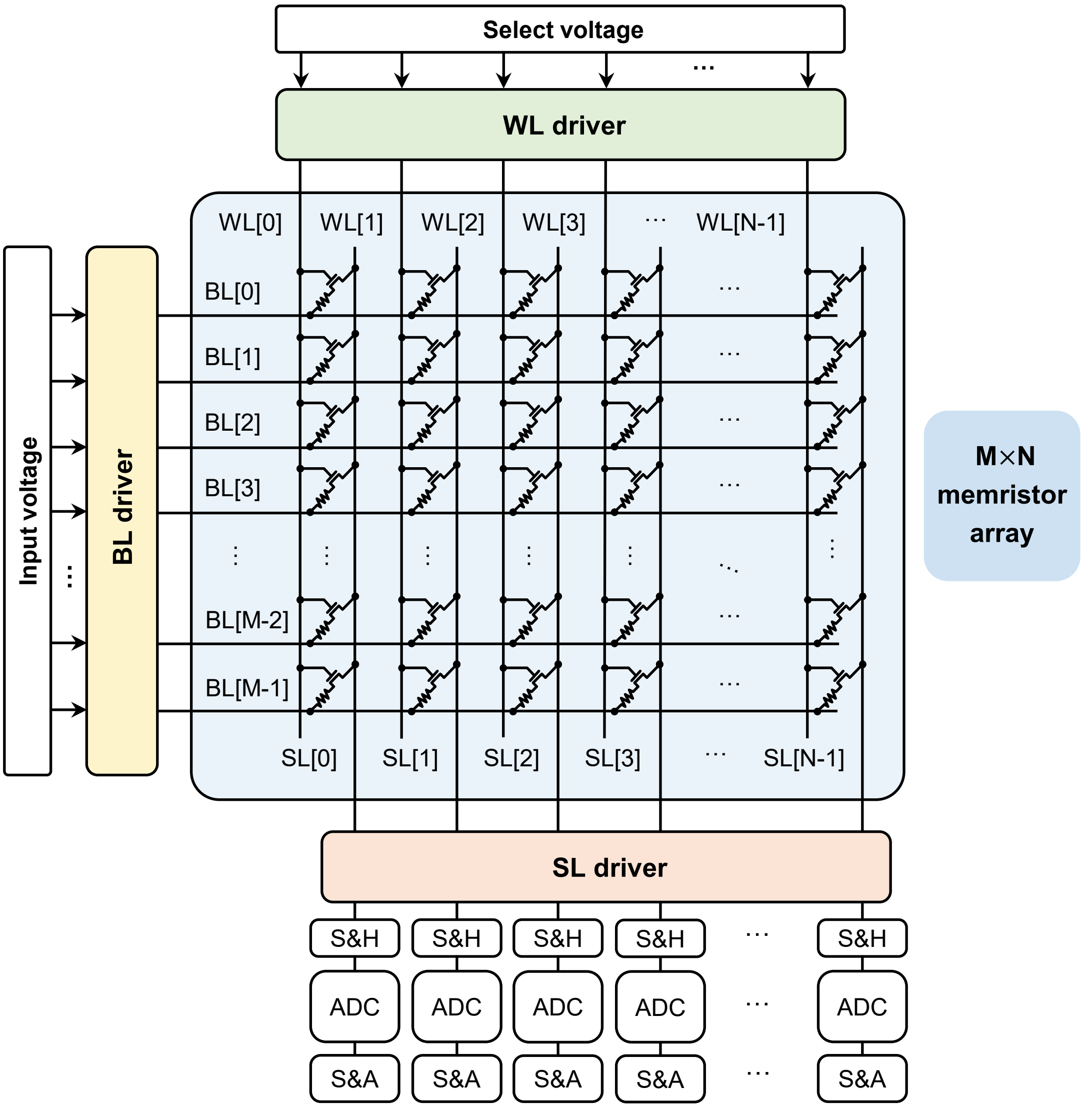

Fig. S13. Macro model of the memristor chips in memCS. The model consists of an M×N memristor array, bit/word/source line (BL/WL/SL) drivers, Sample & Hold (S&H), analog-digital converter (ADC) and Shift & Add (S&A).

Module	Total number	Latency/ns	Energy/pJ
BL driver	384	10.1	31.3
WL driver	384	10.4	
SL driver	384	12.1	
Memristor array	64K	——	342.5
Sample & Hold (S&H)	384	——	0.6
ADC	384	2.0	3891.2
Shift & Add (S&A)	384	10	195.12
Sum	——	44.6	4460.8

Table. S8 Benchmark details of the memristor chips in memCS. Two memristor sub-arrays with size 128×256 and 256×128 are used for the MVM computations in the memCS. The performance of the BL/WL/SL driver and memristor array is inferred from the experimental data of the 128Kb memristor chip. The S&H, ADC and S&A modules are estimated based on reported works [10, 16, 19].

Term	Value
SBC rated power	20 W
SBC energy efficiency	12.8 GOPS/W
SBC Process node	12 nm
Data type	INT8

Table. S9 Configuration table for Milk-V Meles SBC. The latency and energy consumption of the non-MVM components in memCS are estimated using the parameters listed in the table. The results are then normalized to the 130 nm process node for comparison.

Term	Value
PCIe 5.0 power (per channel)	2.5 W
PCIe 5.0 bandwidth (per channel)	32 Gb/s

Table. S10 Configuration table for PCIe 5.0. The latency and energy consumption of the data transmission part in memCS are estimated using the parameters listed in the table. A conservative configuration of eight channels is assumed for the estimation.

**Reconstruction latency (ns/iteration)**			
**MVM**	**Non-MVM**	**Data transmission**	**Total**
44.64	623.62	56.09	724.36
**Energy consumption (μJ/iteration)**			
**MVM**	**Non-MVM**	**Data transmission**	**Total**
0.004	15.27	1.28	16.55

Table. S11 Breakdown of latency and energy consumption for memCS per iteration.

Note 10: Further discussion on performance benchmark

To evaluate the scalability of memCS, we conduct a comparative performance analysis against a GPU-based CS implementation for various input size N. The results for latency and energy consumption are presented in Fig. S14. For the GPU-based CS, both metrics exhibit a quadratic growth trend, scaling approximately as $O(N^2)$. This is primarily attributed to the substantial overhead associated with transferring and processing large-scale matrices. In contrast, memCS leverages in-situ MVM to fundamentally eliminate this $O(N^2)$ increase. Consequently, its overall overhead is dominated by components that scale linearly with the input size N, such as the peripheral array circuits, non-MVM computing, and the transmission of intermediate results between the memristor and digital cores. This crucial difference in scaling behavior leads to a rapidly widening performance gap. At an input size of N=256, memCS already demonstrates a significant 11.22× advantage in latency and a 30.46× advantage in energy consumption. When the input size is quadrupled to N=1024, these advantages become even more pronounced, reaching an impressive 43.36× for latency and 117.50× for energy consumption. These results highlight the superior scalability of memCS for large-scale CS applications.

Furthermore, we discuss the potential of monolithic integration, where the memristor and digital cores are fabricated on a single chip. In such a fully integrated scenario, the physical proximity and increased interconnect bandwidth would render the latency and energy consumption of data transmission between cores negligible compared to the computing overhead of the digital logic itself. Simulating this effect by excluding transmission costs from our evaluation in Note 9 reveals only a modest performance improvement of approximately 1.08× in both latency and energy consumption. The limited gain is because, in our performance evaluation, the digital core's process node was normalized from 12 nm to 130 nm to match the memristor core. This normalization artificially degraded the digital core's energy efficiency, causing the overhead of the digital core itself to dominate the system's overall latency and energy consumption. Therefore, while monolithic integration is a promising direction, its full potential would be unlocked when the process nodes of the memristor and digital cores are matched, and ideally, both are fabricated on a more advanced process.

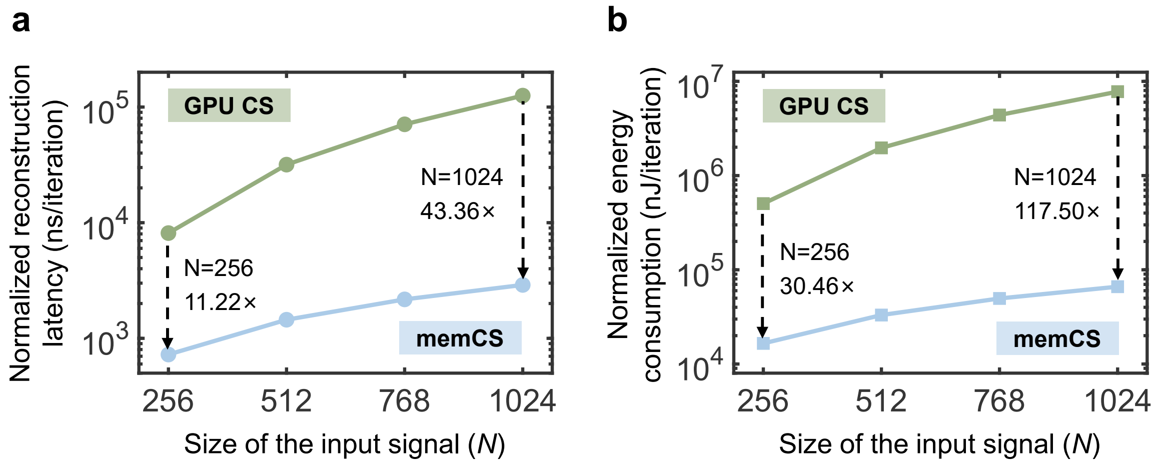

Fig. S14 Performance comparison between GPU-based CS and memCS across different input signal sizes (N). (a) Latency. (b) Energy consumption. Benefiting from in-situ MVM, the latency and energy consumption of memCS scale approximately linearly with the input size ($O(N)$). In contrast, those of the GPU-based CS exhibit a quadratic growth ($O(N^2)$), indicating that the performance advantage of memCS becomes increasingly significant as N grows.

References

1. Zhang W, Yao P, Gao B *et al.* Edge learning using a fully integrated neuro-inspired memristor chip. *Science* 2023; **381**: 1205-11.
2. Zhong Y, Tang J, Li X *et al.* A memristor-based analogue reservoir computing system for real-time and power-efficient signal processing. *Nat Electron* 2022; **5**: 672-81.
3. Qin Q, Gao B, Liu Q *et al.* Hybrid Precoding with a Fully-Parallel Large-Scale Analog RRAM Array for 5G/6G MIMO Communication System. In: *2022 International Electron Devices Meeting* 2022; 33.2.1-.2.4.
4. Candes EJ, Tao T. Near-Optimal Signal Recovery From Random Projections: Universal Encoding Strategies? *IEEE Trans Inf Theory* 2006; **52**: 5406-25.
5. Donoho DL, Johnstone IM. Ideal spatial adaptation by wavelet shrinkage. *Biometrika* 1994; **81**: 425-55.
6. Addison PS. Wavelet transforms and the ECG: a review. *Physiol Meas* 2005; **26**: R155-R99.
7. Stéphane M. CHAPTER 7 - Wavelet Bases. In: Stéphane M (ed.) *A Wavelet Tour of Signal Processing: The Sparse Way (Third Edition)*. Boston: Academic Press, 2009, 263-376.
8. The UCR Time Series Classification Archive. https://www.cs.ucr.edu/~eamonn/time_series_data_2018/ (3 October 2025, date last accessed).
9. Xi Y, Tang J, Gao B *et al.* The Impact of Thermal Enhance Layers on the Relaxation Effect in Analog RRAM. *IEEE Trans Electron Devices* 2022; **69**: 4254-8.
10. Yao P, Wu H, Gao B *et al.* Fully hardware-implemented memristor convolutional neural network. *Nature* 2020; **577**: 641-6.
11. Qin Q, Zhang Q, Gao B *et al.* A Crossbar-wise IR-drop Compensation Schemes for 5G/6G Hybrid Precoding with Highly-parallel Analog RRAM Array. In: *2023 Silicon Nanoelectronics Workshop* 2023; 45-6.
12. NVIDIA Corporation. *NVIDIA H100 Tensor Core GPU*. https://www.nvidia.com/en-us/data-center/h100/ (22 November 2024, date last accessed).
13. Horowitz M. 1.1 Computing's energy problem (and what we can do about it). In: *2014 IEEE International Solid-State Circuits Conference Digest of Technical Papers* 2014; 10-4.
14. Lee H, Chang K-YK, Chun J-H *et al.* A 16 Gb/s/Link, 64 GB/s Bidirectional Asymmetric Memory Interface. *IEEE J Solid-State Circuits* 2009; **44**: 1235-47.
15. Liu Z, Wen Z, Chen B *et al.* A dual-channel half-rate 32 Gb/s, 5.3 pJ/bit SerDes transceiver with 3-tap-FFE and CTLE in 28-nm CMOS for very short reach C2C and C2M interconnection. *Microelectron J* 2025; **159**: 106641.
16. Chan CH, Zhu Y, Sin SW *et al.* A 3.8mW 8b 1GS/s 2b/cycle interleaving SAR ADC with compact DAC structure. In: *2012 Symposium on VLSI Circuits* 2012; 86-7.
17. Milk-V. *Milk-V Meles Embarking on the RISC-V Cosmic Journey*. https://milkv.io/zh/meles (23 December 2024, date last accessed).
18. Choi M-C, Lee S, Roh S *et al.* A 2.5–32 Gb/s Gen 5-PCIe Receiver With Multi-Rate CDR Engine and Hybrid DFE. *IEEE Trans Circuits Syst II Express Briefs* 2022; **69**: 2677-81.
19. Zhang W, Peng X, Wu H *et al.* Design Guidelines of RRAM based Neural-Processing-Unit: A Joint Device-Circuit-Algorithm Analysis. In: *2019 56th ACM/IEEE Design Automation Conference* 2019; 1-6.
